# Building bridges with awe: Exploring underlying mechanisms and moderators of the relationship between awe and prejudice towards sexual minority group members

**DOI:** 10.1111/bjso.12884

**Published:** 2025-04-02

**Authors:** Wang Changcheng, Alice Lucarini, Veronica Margherita Cocco, Kim Dierckx, Loris Vezzali

**Affiliations:** ^1^ Population Research Institute Nanjing University of Posts and Telecommunications Nanjing China; ^2^ Jiangsu High‐Quality Development Comprehensive Evaluation Research Base Nanjing China; ^3^ High‐Quality Development Evaluation Research Institute Nanjing University of Posts and Telecommunications Nanjing China; ^4^ Faculty of Medicine University of Modena and Reggio Emilia Reggio Emilia Italy; ^5^ Department of Developmental, Personality, and Social Psychology University of Ghent Ghent Belgium

**Keywords:** awe, belief in oneness, prejudice, self‐transcendence, sexual minorities

## Abstract

Awe is a self‐transcendent emotion generating a range of benefits at the individual and at the societal level. Yet, research within the domain of intergroup relations is scarce. Across three studies—two experimental and one cross‐sectional (total *N* = 2113)—we explored whether, how and for whom awe is negatively related to prejudice towards sexual minority group members (LGBT individuals) among sexual majority group members (heterosexual people). We found that participants assigned to a nature‐induced awe (vs. control, Study 1; vs. control vs. amusement, Study 2) condition reported lower prejudice towards LGBT people. Moreover, Study 2 shed light on the underlying mechanisms through which nature‐induced awe leads to lower prejudice, demonstrating the parallel mediating role of self‐transcendence and belief in oneness, two constructs related to the promotion of broader group identities, by changing perceptions of the self and the world, respectively. Study 3 revealed that dispositional awe exerted a stronger negative effect on prejudice among heterosexual individuals with less frequent intergroup interactions with LGBT group members. The present investigation provides important insights into the complexity of the relationship between awe and prejudice, revealing that awe could be a powerful tool to reduce prejudice.

## INTRODUCTION

Promoting harmonious relationships between sexual majority and minority group members is a crucial cornerstone in the effort to foster an inclusive society where all individuals can live their lives free from discrimination and prejudice. However, despite the fact that public attitudes towards sexual minority group members have undergone positive changes (Ayoub & Garretson, 2017), people who belong to these groups are nonetheless still the target of prejudice and discrimination, even in relatively tolerant countries (Cramwinckel et al., 2018). In Asia and the Pacific, including China—the country in which we collected data for these studies—sexual minority people are a quite vulnerable social group, with prejudice towards them being widespread (United Nations Development Programme, 2016). This impacts sexual minority group members' quality of life both physically and psychologically; understanding the mechanisms to reduce such prejudice is therefore of paramount importance for developing effective policies and interventions to reduce this social issue, and promote greater acceptance of sexual minority people.

An emerging avenue in research on prejudice reduction towards disadvantaged groups, including sexual minorities, might involve exploring the role of awe, a complex emotion emerging when people face literal or metaphorical vastness (Keltner & Haidt, 2003). So far, however, the benefits of awe identified by research were mainly limited to the individual and the interpersonal level. For instance, awe was associated with reduced stress and increased life satisfaction (Bai et al., 2021), and it was found to promote prosocial behaviour (Jiang & Sedikides, 2022; Perlin & Li, 2020; Piff et al., 2015). Based on preliminary evidence (Luo et al., 2022; Lv et al., 2024; Stamkou et al., 2023; Stell, 2018), we speculate that awe might also have beneficial effects at the intergroup level: it is possible that awe is negatively associated with prejudice towards disadvantaged groups—such as people belonging to sexual minorities. The present investigation consists of two experimental and one correlational study and undertakes to explore for the first time whether, how and for whom awe is (more strongly) negatively related to prejudice against sexual minority group members.

### Awe and prejudice

Awe is a predominantly positive emotion produced by the appraisal of stimuli that are vast and transcend current frames of reference (Bonner & Friedman, 2011; Keltner & Haidt, 2003), including natural landscapes, the cosmos, spiritual and religious experiences but also human achievements, such as revolutionary theories, art and music. Although there is a paucity of research addressing awe in prejudice reduction research—with no direct evidence specifically linking it to prejudice against sexual minority group members—multiple cues suggest that awe has the potential to diminish prejudice.

First, awe belongs to a taxonomy of emotions called “self‐transcendence emotions,” which can encourage individuals to shift the focus from the self to others (Stellar et al., 2017). Other emotions in this category include, for instance, gratitude and compassion (DeSteno et al., 2016). All self‐transcendent emotions are other praising, arising out of other‐focused appraisals (Algoe & Haidt, 2009); therefore, self‐transcendence emotions lead individuals towards greater care for others' welfare (Stellar et al., 2017). Despite the shared similarities with other emotions in the same taxonomy, we argue that awe might stand out in the promotion of the welfare of groups that are systematically stigmatized. This might depend on the fact that, unlike other self‐transcendence emotions, awe has the unique capacity to foster a broader, non‐egocentric viewpoint that not only favours an increased focus on others but also a “big‐picture” perspective on life itself (Pan & Jiang, 2023). Indeed, facing stimuli that are vast and transcend the current frames of reference requires accommodation processes allowing existing mental schemata to assimilate new experiences (Keltner & Haidt, 2003), leading to profound changes in how people see themselves and the world (Chirico & Yaden, 2018). For instance, stimuli eliciting awe contribute to a deeper connection with humanity and the planet (Yaden et al., 2016), increased use of collective language (Goldy et al., 2022), more inclusive self‐descriptions (Shiota et al., 2007) and global identification processes (Seo et al., 2023). In other words, by enabling the shift to a more inclusive common identity (Gaertner & Dovidio, 2000), awe could have the potential of breaking group boundaries and reducing prejudice, something which other self‐transcendence emotions might fail to do, as their emergence is often dependent on group‐related factors such as perceived similarity (see DeSteno, 2015).

Second, while awe is not the only self‐transcendent emotion that can have powerful implications at the intergroup level, its unique characteristics could open up new and powerful pathways for prejudice reduction. Both kama muta (Blomster Lyshol et al., 2023) and moral elevation (Engels et al., 2024) were found to promote positive intergroup outcomes, and they share with awe the ability to foster a sense of connection and communality with others (Pizarro et al., 2021). However, despite these similarities, the three emotions are elicited by and operate through distinct stimuli and mechanisms, potentially leading to different avenues for prejudice reduction. Kama muta is elicited by perceptions of relational closeness (Schubert et al., 2018); moral elevation is a reaction to witnessing moral virtue or beauty in others' behaviour (Haidt, 2000). Differently, awe is elicited by perceptions of vastness that emerge from universally accessible stimuli, like nature or space, extending beyond relational and moral contexts. This peculiarity of awe, combined with the unique cognitive process of accommodation that it induces, could favour an even more encompassing view of humanity and the world, resulting in significant outcomes at the intergroup level.

Third, a growing number of studies demonstrated that awe is conducive to the solution of relevant social problems (e.g. climate change inaction; Wang et al., 2022) and may therefore also be implicated in relevant social issues such as intergroup disparities and associated discrimination towards certain groups. Most germane to our investigation, awe has already been associated with a range of prejudice‐related outcomes. It was found that inducing awe reduces blatant dehumanization towards obese people, and its effect is mediated by increased common ingroup identity (Lv et al., 2024). The beneficial effects of awe in terms of increased prosocial behaviours (see Piff et al., 2015) seem to also apply within an intergroup framework: Stamkou et al. (2023) found that Dutch children (ethnic majority) who watched awe‐inducing videos were more willing to behave prosocially towards refugees (ethnic minority outgroup). Moreover, Stell (2018) found that a heightened sense of awe is causally related to a lower level of implicit gender and ethnic stereotypes that cause intergroup suffering (but contradictory findings have been reported for African‐American people; Dale et al., 2020). Additionally, preliminary evidence suggests that both experimentally induced and dispositional awe are consistently negatively associated with AIDS‐related stigma (Luo et al., 2022).

Finally, awe also has an effect on personality and cognitive factors related to prejudice. As previously anticipated, awe can cultivate a more open mindset and worldview by challenging existing schemata and promoting a broad, non‐egocentric perspective on the self. This is consistent with research findings linking dispositional awe to greater openness to experience (Shiota et al., 2006) and curiosity (Anderson et al., 2020), respectively a personality trait and an individual disposition that are relevant for prejudice reduction (Jackson & Poulsen, 2005; Lucarini et al., 2023). On a similar note, when people experience awe, they are less reliant on heuristic‐driven information processing (Griskevicius et al., 2010), and reduced heuristic thinking has been associated with decreased dehumanization towards multiple stigmatized groups (Prati et al., 2015, Study 2). Finally, dispositional awe was also found to be negatively related to the need for cognitive closure (Shiota et al., 2007), another relevant individual disposition that (positively) correlates with prejudice (Roets & Van Hiel, 2011).

Summarizing, while preliminary research on the effects of awe at the intergroup level is growing, several key aspects are yet to be disentangled. First of all, prior studies focused primarily on constructs related to prejudice but not overlapping with it, such as dehumanization (Lv et al., 2024) or stigma (Luo et al., 2022). In contrast, our studies provide a direct test on the effects of awe on intergroup attitudes, employing different measures to assess attitudes towards our target outgroup. Second, in our studies, we take into account a group, that is sexual minority individuals, which has not been the focus of prior research and is particularly relevant given the national context of our investigation. Third, beyond a direct test, our studies aim to provide a comprehensive framework on the effect that awe exerts on prejudice, exploring how and for whom awe has a prejudice‐reducing effect.

### Underlying mechanisms: The mediating role of self‐transcendence and belief in oneness

As discussed in the previous paragraph, experiences of awe lead to profound changes in how people see themselves and the world, arguably by fostering social identification processes that allow individuals to adapt to broader group identities (see also Ejova, 2019). On the one side, awe can exert a transformative impact on the self in terms of reduced self‐salience (Jiang et al., 2024). From a psychological perspective, we can define this phenomenon as self‐transcendence, which is conceptualized both as the process of expanding beyond one's self‐boundaries (Aron et al., 1992; Aron & Aron, 1997; Reed, 2013) and as the outcome of this expansion (Garcia‐Romeu, 2010; Reed, 1991). Indeed, as discussed above, awe is a self‐transcendent emotion that helps us free from mundane concerns and turns our minds outward to vaster entities, thus encouraging us to transcend our day‐to‐day agendas and limits, as well as the self (Bonner & Friedman, 2011; Jiang et al., 2018; Shiota et al., 2017). This allows a greater sense of identification with others and an increased sensitivity to the external world (Haidt, 2003; Halstead & Halstead, 2004; Pappas & Friedman, 2007; Shiota et al., 2007).

The causal relation between awe and self‐transcendence has received large empirical support, with higher levels of awe associated with increased self‐transcendence (Dai & Jiang, 2023; Jiang & Sedikides, 2022). Moreover, the shift from our own momentary needs and concerns entailed in the experience of self‐transcendence is likely to make us accept others as equals, in line with empirical research showing that high levels of self‐transcendence are associated with a more accepting attitude towards outgroup members (Schwartz, 2010).

On the other side, awe not only impacts the self by diminishing self‐importance (i.e. self‐transcendence), it also affects how we see the world itself. As discussed in the previous paragraph, through awe we gain a “bigger‐picture” perspective on life because feelings of awe allow us to process a larger and complex reality. This could be the spark to initiate a transformative process regarding perceptions of the integration of the self with others, as well as with the surrounding environment (Chirico & Yaden, 2018). By affecting people's mental schemas, awe could enable them to embrace new beliefs and worldviews. A possible outcome of this path is the increased belief that everything that exists is part of an all‐encompassing fundamental whole (i.e. belief in oneness; Diebels & Leary, 2019).

Though it has been quite a prevalent concept in many cultures and within a variety of philosophical, religious, spiritual and scientific perspectives (Capra, 1975; DeLuca, 2003; Happold, 1963; James, 1902; Lande, 1971; Molloy, 2002; Schaffer, 2016), belief in oneness became the subject of empirical research in psychological sciences just recently. Relatedly, the relationship between awe and belief in oneness has been poorly investigated; however, the literature suggests that people can come to believe that everything is one as the result of a personal mystical experience (Hood Jr., 2016), as the experience of awe could be.

From a psychological perspective, belief in oneness boasts vital implications for our attitudes towards other people, including outgroup members. People who believe in oneness conceptualize themselves in terms of their relations with others and incorporate them into their own sense of self (DeCicco & Stroink, 2007; Leary et al., 2008), which has been shown to make people feel more connected to both close and distant others (Diebels & Leary, 2019). Most relevant to the present work, endorsing an increased belief in oneness might allow people to expand the boundaries of their own group by drawing outgroup members into their circle of concern (Gaertner et al., 1993; Gaertner & Dovidio, 2000; McFarland et al., 2013; Monroe, 1996). This is consistent with the idea that perceiving a shared, higher‐level identity with people from other social groups has relational and societal benefits (Gaertner et al., 1999). Consistent with the evidence presented above, the cultivation or enhancement of belief in oneness is likely to result in lower prejudice.

Summarizing, we argue that awe could be associated with reduced prejudice by promoting broader, more inclusive group identities and that this occurs, at least partially, via two key processes: one related to the perception of the self, that is self‐transcendence, a largely investigated process in awe literature; the other, related to the perception of the world itself, that is belief in oneness, whose relationship with awe has yet to be clearly disentangled.

### Conditioning factors: The moderating role of intergroup contact

Intergroup contact has long been advocated as one of the most effective prejudice reduction strategies (Allport, 1954). After nearly 70 years of research conducted across diverse settings and social groups, scholars generally agree with Gordon Allport's contact hypothesis that positive interactions between ingroup and outgroup members can lessen intergroup prejudice (Pettigrew & Tropp, 2006; Ülger et al., 2018). These results hold strong and consistent within a wide range of intergroup contexts, including those involving contact between sexual minority and sexual majority group members (Cramwinckel et al., 2021; Kanamori et al., 2022; Lissitsa & Kushnirovich, 2021; Vezzali et al., 2017). For instance, in a large multi‐sample study with participants from 77 countries across North America, South America, Europe, Africa, Asia and Oceania, Earle et al. (2021) found that having contact with members of a sexual minority group was associated with greater support for their rights. More recently, a meta‐analysis on prejudice reduction by Paluck et al. (2021) reported a reliable (although modest) effect of contact on reducing prejudice towards sexual minority groups (*d* = 0.22).

In the previous paragraphs, we argued that awe could enable the shift to a more inclusive common identity (Gaertner & Dovidio, 2000; see Lv et al., 2024), thus breaking group boundaries and reducing prejudice and that this might be, at least partially, conveyed by two processes related to changes in perception of the self and of the world, that are self‐transcendence and belief in oneness. This is something that awe might share with contact: for instance, when members of two different social groups co‐operatively interact, more harmonious relationships are likely to emerge, at least in part because this favours the development of a common ingroup identity (Gaertner et al., 1993). In other words, despite being two clearly distinct phenomena, contact and awe could lead to the same outcome, namely changing perceptions of group boundaries and favouring a shift from an “us vs. them” to a more inclusive “we” perspective.

Summarizing, given that intergroup contact is relevant to prejudice reduction, also because of its ability to promote common ingroup identity—something which, arguably, it shares with awe and its underlying mechanisms—it is plausible that the effect of awe depends, at least in part, on whether individuals had interactions with sexual minority group members. In other words, intergroup contact experiences may be a conditioning factor that explains inter‐individual variability in the association of awe with prejudice. Specifically, it is possible that awe and contact operate together so that the effects of awe emerge more strongly among individuals who have low levels of contact; in contrast, for those who have high levels of contact, re‐categorization processes and outgroup attitudes might be based mainly on their direct experiences (that is, contact) rather than on awe (for a similar rationale applied to indirect contact or the application of social norms, see for example Di Bernardo et al., 2023; White et al., 2021). We argue that the same rationale applies to the relationship of awe with self‐transcendence and belief in oneness. As awe promotes accommodation processes challenging current mental schemata and changing people's view of themselves and the world, intergroup contact can be a liberalizing agent (Hodson et al., 2018), promoting changes in socio‐cultural and cognitive elements of human experience (Fuochi et al., 2024).

### The present research

The current investigation delves into the issue of whether, how and for whom awe is related to lower prejudice towards sexual minorities among sexual majority group members. Across two experimental studies and one correlational study, we directly examine the relationship between awe and prejudice towards sexual minority group members (referring to LGBT individuals, in all three studies).

First of all, we experimentally tested the causal relationship between awe and prejudice towards members of sexual minority groups, in samples of sexual majority individuals (Studies 1 and 2). Then, we attempted to identify the underlying mechanisms through which awe is associated with less prejudice towards members of a sexual minority group, by testing the mediating role of self‐transcendence and belief in oneness (Study 2). These two studies are primarily aimed at providing causal evidence for the effects of awe. Finally, to gain a broader picture, in a correlational study, we also took into account the moderating role of intergroup contact in the relationship between awe and prejudice, mediated by self‐transcendence and belief in oneness (Study 3). Using a correlational design, after having obtained causal evidence with the first two studies, allowed us to replicate results with a different design and a different measure of awe (and specifically, capturing dispositional awe), increasing the generalizability of our findings. Moreover, it allowed us to include an ecological measure of contact based on participants' lived experiences. This test helps address the question of whether higher levels of awe invariably link with lower prejudice against members of a sexual minority group.

In summary, building on previous research outlined above, we hypothesise that:

Participants assigned to an awe‐inducing condition will report lower prejudice towards sexual minority group members compared to those assigned to control (Studies 1 and 2) and amusement‐inducing (Study 2) conditions.

The reduction in prejudice towards sexual minority group members observed in the awe‐inducing condition will be explained, at least partially, by increased feelings of self‐transcendence and belief in oneness.

Intergroup contact will moderate the relationship between awe and prejudice. More specifically, we predict that the effects of awe will emerge more strongly among people who have less frequent contact experiences, compared to those who have numerous intergroup encounters. Consistent with the claim that awe and contact can work as complementary strategies in promoting broader group identities, we hypothesize the same moderation effect on self‐transcendence and belief in oneness.

Importantly, to ensure that the hypothesized association of awe with prejudice against sexual minority group members is not simply part of a more general tendency for positive states to reduce prejudice and in line with prior existing research on awe (Jiang & Sedikides, 2022; Piff et al., 2015), in all three studies we statistically controlled for a range of other emotions, outlined below.

The studies reported in this manuscript, all conducted in China, involve human participants and were approved by the Research Ethics Committee of the University of Nanjing University of Posts and Telecommunications. All procedures of these studies are in accordance with the 1964 Helsinki Declaration and its later amendments or comparable ethical standards. Participants provided informed consent in all studies. All studies were conducted in a major Eastern city in China. Data, syntaxes and materials of all studies are openly available at https://osf.io/guwp3/?view_only=c7880ee430eb4c418a217f346e6d13d5.

## STUDY 1

Study 1 aimed to ascertain whether higher levels of awe are causally related to lower prejudice towards sexual minority group members in a sample of individuals belonging to the sexual majority group. To do so, we designed a laboratory experiment where participants were either exposed to a nature awe‐inducing video or a control video, eliciting a neutral state.

### Method

#### Participants

The target of Study 1 (also applies to other studies) were participants who self‐identified as heterosexual. As a result, a primary eligibility criterion for participation in Study 1 was identifying as heterosexual (“Please answer anonymously in which of the following categories you self‐categorize: A. heterosexual B. LGBT C. Other”); people who did not identify as heterosexual could not participate in the experiment. A total of 433 participants recruited in China voluntarily took part in Study 1, in exchange for monetary compensation (5 RMB, corresponding approximately to 0.6 Euros). As for exclusion criteria, 22 participants were excluded from subsequent statistical analysis: 5 participants did not comply with instructions (i.e. started answering the questionnaire before they finished watching the video), 11 participants failed an attention check embedded within the questionnaire, and additional 6 participants reported technical issues while watching the video. This left a final sample of 411 participants (209 women) aged between 20 and 43 years (*M* = 27.32, *SD* = 6.17). All participants included in the analyses reported being heterosexual.

Results of a sensitivity power analysis run on G*Power (Faul et al., 2007) for a one‐way between‐subjects design with two conditions revealed that with *α* = .05 and 1−*β* = 0.80, the minimum detectable effect size was *f* = 0.14, which can be considered a small effect (Cohen, 1988; refer to the Supporting Information for a more detailed comment on the results of the power analysis of Study 1).

#### Procedure

The study was advertised via flyers distributed among the population and one research assistant was responsible for data collection. Participants were informed that they were taking part in an in‐lab study on emotional experiences and social interactions. All of them engaged in the experiment individually: each participant arrived at the lab at separate times and completed the study alone, without interacting with other participants. Overall, the study lasted 15–20 min. First of all, upon arrival to the lab, participants were welcomed and seated in individual testing cubicles where they were provided with informed consent forms and filled in demographic information. Before exposure to either the experimental or the control group, participants were provided with a definition of awe—“Awe is a predominantly positive emotion produced by the appraisal of stimuli that are vast and transcend current frames of reference (Bonner & Friedman, 2011; Keltner & Haidt, 2003), including natural landscapes, the cosmos, spiritual and religious experiences, but also human achievements, such as revolutionary theories, art, and music”; see OSF for the questionnaire—to ensure conceptual clarity and consistent interpretation among participants, similar to previous research (Bai et al., 2017). Then, they were randomly assigned to watch one out of two videos lasting 302 s, using the provided headsets (see OSF for the videos), either eliciting awe (experimental condition, *N* = 207) or a neutral state (control condition, *N* = 204). In the experimental condition, participants watched the video designed and found to be effective in eliciting awe (Hornsey et al., 2018, adaptation by Jiang & Sedikides, 2022), which showed the comparative sizes of the Earth and other celestial bodies. Earth is presented in comparison with bigger celestial bodies, from other planets to stars, highlighting how even stars are just tiny dots in the universe. In the control condition, participants watched a video displaying an ordinary street scene in Guangzhou, China. A walking camera operator (not visible in the video) filmed various areas of the city, including streets, buildings and local shops. Several passersby appear in the video, but none of them was the focus of the shot; they were simply captured as part of the surrounding environment. Afterward, participants were administered an online questionnaire, via computer. At the end of the experiment, they were thanked, debriefed and rewarded.

#### Measures

##### Awe

Participants rated the extent to which they were feeling awe, together with a list of other positive and negative emotions, namely anger, pride, disgust, fear, happiness and sadness, employing a 7‐point Likert scale ranging from 1 (*not at all*) *to* 7 (*extremely*). This is consistent with previous research controlling for a range of positive and negative emotions in subsequent statistical analyses (Jiang & Sedikides, 2022; Piff et al., 2015); in line with previous research, scores for these emotions were not aggregated.

##### Prejudice

To assess prejudice towards sexual minority group members, participants filled in an adapted version of the semantic differential scale (Wright et al., 1997). Participants were asked to report how they feel about LGBT individuals, by employing five bipolar adjective pairs separated by a 7‐point scale (Wright et al., 1997; “warm‐cold”; “negative–positive” – reverse coded; “friendly‐hostile”; “suspicious‐trusting– reverse coded”; “respect‐contempt”; *α* = .92). Items were combined so that higher scores indicated more prejudice.

### Results and discussion

To test whether the awe manipulation (vs. control) was effective, we performed a one‐way ANOVA. Results showed that participants' self‐reported feelings of awe significantly differed between conditions, *F*(1, 409) = 164.47, *p* < .001, $\eta_{p}^{2}$ = .29. Participants who watched the awe‐inducing video experienced stronger feelings of awe (*M* = 4.51, *SD* = 1.43), compared with those who watched the neutral video (*M* = 2.56, *SD* = 1.64), indicating that the manipulation was effective. Results were replicated by controlling for anger, pride, disgust, fear, happiness and sadness, *F*(1, 403) = 167.62, *p* < .001, $\eta_{p}^{2}$ = .29.^1^

Following the same rationale, we ran a one‐way ANOVA to test the effect of the awe‐inducing condition (vs. control) on prejudice towards LGBT individuals. Results showed a significantly different degree of prejudice depending on the experimental condition, *F*(1, 409) = 132.03, *p* < .001, $\eta_{p}^{2}$ = .24. In line with our hypothesis, participants who watched the awe‐inducing video reported a lower level of prejudice (*M* = 2.40, *SD* = 0.85) than participants in the control condition (*M* = 3.43, *SD* = 0.97). Again, having anger, pride, disgust, fear, happiness and sadness controlled for, results were replicated, *F* (1, 403) = 136.08, *p* < .001, $\eta_{p}^{2}$ = .25.

Findings of Study 1 provide—to our knowledge, for the first time—preliminary experimental evidence that inducing awe, compared to a neutral state, results in lower prejudice towards sexual minority group members, in a sample of participants belonging to the sexual majority group. Study 2 aims to take a step further by conceptually replicating the results of Study 1 and investigating the possible underlying mechanisms.

## STUDY 2

In this second, experimental study we built upon the findings of Study 1. First, we aimed to replicate the effect of awe‐induction on prejudice in a different sample. In Study 2, we also added a third condition, namely an amusement induction condition. Importantly, we chose amusement as a comparison to awe for several reasons. First of all, the two share some similarities: they are both positive emotions elicited by an incongruence between one's expectations (or default schema) and experience (Morreall, 1989). Second, amusement‐inductions are commonly employed to induce general positivity and can be reliably elicited via video clips (Algoe & Haidt, 2009; Bartlett & DeSteno, 2006). Third, amusement has been widely used in prior research to control for general positivity's effect when inducing awe (Piff et al., 2015; Stellar et al., 2018; Valdesolo & Graham, 2014; Van Cappellen & Saroglou, 2012; Yuan et al., 2024). Fourth, it is worth noting that in our manipulations we chose to elicit awe and amusement via video clips portraying images related to nature, that is natural landscapes in the awe conditions and nonhuman beings (animals) in the amusement condition. That allows us to ascertain the specific effects of awe on prejudice, beyond the mere exposure to nature‐related contents.

To further generalize our findings and in line with the methodology adopted in prior research, in Study 2 we used different videos as stimuli, widely employed in the literature on awe (Dai & Jiang, 2023; Piff et al., 2015; Valdesolo & Graham, 2014; Yuan et al., 2024). Second, we sought to investigate the psychological processes underlying the relationship between awe and prejudice. Specifically, we explored the mediating role of self‐transcendence and belief in oneness in the relationship between awe and prejudice.

### Method

#### Participants

As for Study 1, self‐identifying as heterosexual was a primary eligibility criterion for participation in Study 2 (“Please answer anonymously in which of the following categories you self‐categorize: A. heterosexual B. LGBT C. Other”); those who did not identify as heterosexual could not participate in the experiment. A total number of 525 participants recruited in China voluntarily took part in an in‐lab experiment, in exchange for a monetary reward (6 RMB, corresponding to approximately 0.8 Euros). As for exclusion criteria, 10 participants who failed an attention check were excluded from all analyses; additionally, 1 participant reported health conditions (coughing symptoms and headache) while doing the experiment, with these symptoms preventing them from completing the study, thus they were also excluded from the analyses. The final sample included 514 participants, aged from 18 to 45 years old (271 women, *M* = 26.64, *SD* = 7.39). As in Study 1, all participants included in the study reported being heterosexual.

**FIGURE 1:**
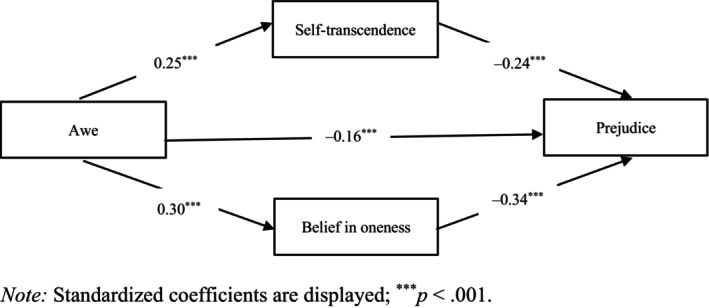
Mediation model for Study 2. Standardized coefficients are displayed; ***p < .001.

Results of a power sensitivity analysis run on G*Power (Faul et al., 2007) for a one‐way between‐subjects design with three conditions revealed that with *α* = .05 and 1−*β* = 0.80, the minimum detectable effect size was *f* = 0.14, which can be considered a small effect (Cohen, 1988; refer to the Supporting Information for a more detailed comment on the results of the power analysis of Study 2). Moreover, we ran a Monte Carlo power sensitivity analysis (Schoemann et al., 2017; Thoemmes et al., 2010). The selected model was two parallel mediators, with 10,000 replications, 20,000 Monte Carlo draws per replication, a 95% confidence level and 1234 as random seed. The simulation was based on the standardized coefficient emerging from the mediation model tested in Study 2 (*see* Figure 1). The analysis indicated that with 514 participants, we had 96% and >99% statistical power to detect the mediation of self‐transcendence and belief in oneness, respectively.

#### Procedure

To recruit participants for Study 2, we followed the same sampling procedure employed in Study 1: the study was advertised via flyers distributed among the general population and one research assistant was responsible for data collection. As in Study 1, participants were informed that they were taking part in a study on emotional experiences and social interactions. Each participant completed the experiment individually, with a duration between 15 and 20 min including the same passages employed in Study 1. Once they arrived in the lab, they signed the informed consent form and provided demographic information in individual testing cubicles. Again, consistent with Study 1, as well as with previous research, participants were provided with a definition of awe (same as Study 1; see OSF for the questionnaire). Then, they were randomly assigned to watch one out of three videos, using the provided headsets (see OSF for the videos), which had been previously used and found to be effective in prior research. As in Study 1, one of these videos aimed to elicit awe, another aimed to induce a neutral state and the third video—newly introduced for this study—aimed to induce feelings of amusement (Piff et al., 2015; Valdesolo & Graham, 2014). Participants in the awe condition (*N* = 172) were exposed to a 185 s video featuring nature clips sourced from the BBC's Planet Earth series. The clips portrayed panoramic shots of scenic vistas, mountains, plains, forests and canyons (Piff et al., 2015; Valdesolo & Graham, 2014; Yuan et al., 2024). Participants in the amusement condition (*N* = 170) watched a 189 s video of animals in their natural habitats acting in funny ways (Piff et al., 2015). Participants in the control condition (*N* = 172) watched a 181 s video displaying an ordinary street scene in Guangzhou, China, which is the same that we used in Study 1 (Jiang & Sedikides, 2022), but it was shortened by removing the first 2 minutes. This change was made to match the length of the videos used in the awe and amusement conditions.

After watching the videos, participants completed a series of measures, detailed below, in an online questionnaire, via computer. Finally, when they finished the questionnaire, participants were debriefed, thanked and rewarded.

#### Measures

To assess awe, other positive and negative emotions, as well as prejudice (*α* = .92), we employed the same measures as in Study 1. Additionally, we assessed:

##### Amusement

Amusement was assessed together with awe and the filler positive and negative emotions. Participants rated the extent to which they were amused, employing a 7‐point Likert scale ranging from 1 (*not at all*) to 7 (*extremely*).

##### Self‐transcendence

Self‐transcendence was measured using four items developed by Jiang et al. (2018). Participants were asked to express their level of agreement with each item (e.g. “I want to find answers to some universal spiritual questions”) on a 7‐point scale ranging from 1 (*strongly disagree*) to 7 (*strongly agree*). Higher scores reflect a stronger sense of self‐transcendence (*α* = .87).

##### Belief in oneness

We administered the Belief in Oneness Scale to assess the extent to which participants felt it easy to believe each of five statements, reflecting the tendency to believe that all existing entities are part of an all‐encompassing fundamental whole (Diebels & Leary, 2019; e.g. “Everything is composed of the same basic substance, whether one thinks of it as spirit, consciousness, quantum processes, or whatever”; 1 = *very difficult for me to believe this is true*, 5 = *very easy for me to believe this is true*). Higher scores denote a strengthened belief in oneness (*α* = .89).

At the end of the experiment, participants were debriefed, thanked and rewarded.

### Results and discussion

First of all, to verify the effectiveness of our manipulation, we tested whether the awe‐inducing condition elicited more awe compared to the control and the amusement‐inducing conditions. Results were consistent with Study 1: a one‐way ANOVA yielded a significant difference among the conditions, *F*(2, 509) = 133.79, *p* < .001, $\eta_{p}^{2}$ = .35. Post‐hoc Bonferroni‐corrected comparisons showed that participants in the awe‐inducing condition reported feeling significantly more awe (*M* = 4.56, *SD* = 1.57) than those in the amusement (*M* = 1.89, *SD* = 1.46, *p* < .001) or in the control condition (*M* = 2.15, *SD* = 1.92, *p* < .001), while no significant difference was observed between the latter two groups (*p* = .46). Results did not change when controlling for anger, pride, disgust, fear, happiness and sadness, *F*(2, 503) = 133.20, *p* < .001, $\eta_{p}^{2}$ = .35. These findings support the effectiveness of our manipulation. As a further test, we ran the same analysis to see whether perceived amusement was higher in the amusement (vs. awe vs. control) condition. Results showed that participants' perceived amusement significantly differed between conditions, *F*(2, 509) = 203.47, *p* < .001, $\eta_{p}^{2}$ = .44. Post‐hoc Bonferroni‐corrected comparisons showed that participants in the amusement‐inducing condition reported feeling significantly more amusement (*M* = 2.46, *SD* = 1.20) than those in the awe (*M* = 1.09, *SD* = 0.37, *p* < .001) or in the control condition (*M* = 1.05, *SD* = 0.24, *p* < .001), while no significant difference was observed between the latter two groups (*p* = 1.00). These findings provide further support for the idea that increases in awe are distinct from a general rise in positivity (see Footnote 1).

Results of Study 1 were replicated for prejudice: Participants reported a significantly different degree of prejudice depending on experimental condition, *F*(2, 506) = 68.91, *p* < .001, $\eta_{p}^{2}$ = .21. Post‐hoc Bonferroni‐corrected comparisons revealed that participants in the awe condition reported less prejudice (*M* = 2.54, *SD* = 1.09) than those in the amusement (*M* = 3.46, *SD* = 0.67, *p* < .001) and control (*M* = 3.67, *SD* = 1.02, *p* < .001) conditions, whereas the latter two conditions did not significantly differ, *p* = .12. This result held when controlling for anger, pride, disgust, fear, happiness and sadness, *F*(2, 500) = 71.58, *p* < .001, $\eta_{p}^{2}$ = .22.

We ran the same analyses for self‐transcendence and belief in oneness. Results yielded a significant main effect of awe on self‐transcendence, *F*(2, 505) = 32.86, *p* < .001, $\eta_{p}^{2}$ = .12, also when controlling for anger, pride, disgust, fear, happiness and sadness, *F*(2, 499) = 34.10, *p* < .001, $\eta_{p}^{2}$ = .12. Post‐hoc Bonferroni‐corrected comparisons showed that participants assigned to the awe condition (*M* = 4.00, *SD* = 1.44) exhibited a stronger sense of self‐transcendence than those in the amusement (*M* = 3.05, *SD* = 0.81, *p* < .001) and control (*M* = 3.08, *SD* = 1.32, *p* < .001) conditions, while no significant difference in self‐transcendence was observed between amusement and control groups, *p* = 1.00.

As for belief in oneness, we found the same pattern of results, *F*(2, 504) = 56.81, *p* < .001, $\eta_{p}^{2}$ = .18. Again, this was true also when controlling for anger, pride, disgust, fear, happiness and sadness, *F*(2, 498) = 55.12, *p* < .001, $\eta_{p}^{2}$ = .18. In line with previous results, post‐hoc Bonferroni‐corrected comparisons revealed that, compared to the amusement (*M* = 2.52, *SD* = 0.68, *p* < .001) and control (*M* = 2.32, *SD* = 0.98, *p* < .001) conditions, participants assigned to the awe condition reported a stronger belief in oneness (*M* = 3.27, *SD* = 0.91), while no significant difference was found when comparing the amusement and control conditions, *p* = .13.

Finally, to test whether the awe condition is associated with lower prejudice via self‐transcendence and belief in oneness, we performed a bootstrapped parallel mediation analysis with 10,000 resamples using SPSS PROCESS Model 4 (Hayes, 2018; Preacher & Hayes, 2004, 2008); 95% confidence intervals were used to determine statistical significance of the standardized effects. Figure 1 illustrates the mediation model and provides path coefficients of the associations among the variables. As for indirect effects, we found support for a path where the awe condition (in contrast to the amusement and control conditions; coded as awe = 2, amusement = −1, control = −1) predicted less prejudice via heightened self‐transcendence as well as greater belief in oneness; the indirect effects were both significant, *b*_self‐transcendence_ = −.06, *SE* = 0.02, CI [−0.09, −0.03]; *b*_belief in oneness_ = −.10, *SE* = .02, CI [−0.14, −0.07].^2^

Study 2 provided a conceptual replication and an extension of the findings from Study 1. More specifically, we provided further support (a) that inducing awe, compared to amusement and to a neutral state, results in lower prejudice towards members of a sexual minority group; and (b) that this effect is not imputable to a more general positive emotional state (that is amusement), rather it specifically depends on participants' increased feelings of awe. Additionally, our findings support the claim that self‐transcendence and belief in oneness exert a mediating effect on the relationship between awe and prejudice.

So far, the results of the first two studies answered the question of whether and how people experiencing awe, compared to other emotional states, report less prejudice towards sexual minority group members. However, it is worth noting that in Studies 1 and 2 we did not take into account inter‐individual differences in exposure to sexual minorities; that is, we did not gauge participants' intergroup contact experiences, which are widely acknowledged as one of the most effective prejudice reduction strategies (Allport, 1954). Building upon this, in Study 3, we sought not only to further replicate previous findings but also to fill this gap and understand *for whom* these effects are larger, by investigating the potential moderating role of intergroup contact. Additionally, in Study 3 we also employed a different and specific measure to address attitudes towards sexual minority people, providing a more tailored assessment of this construct. Finally, to further generalize our findings, rather than experimentally inducing awe, we included a dispositional measure of awe.

## STUDY 3

Study 3 aims to test whether and for whom higher levels of awe are more strongly associated with lower prejudice towards sexual minority group members. Specifically, we hypothesized a potential moderating role of contact in the relationship between awe and prejudice, also taking into account the—previously demonstrated—mediating role of self‐transcendence and belief in oneness. That is, we expected that, for those individuals reporting lower levels of intergroup contact, awe would be more strongly associated with lower prejudice (via increased self‐transcendence and belief in oneness). To test this hypothesis, we designed a correlational study where participants completed a list of measures assessing awe, contact, prejudice, self‐transcendence and belief in oneness. After having obtained causal evidence for the role of awe in Studies 1 and 2, this correlational study allowed us to replicate findings with a different design. Crucially, it allowed us to include a measure of dispositional awe, which can increase the generalizability of findings across different types of awe (induced vs. dispositional). It also allowed us to include an ecologically valid measure of contact based on participants' real‐life contact experiences. Given that our rationale is based on the idea that the effect of awe depends on the fact that individuals had or had not personal experiences of contact, we relied on a measure of contact quantity. Importantly, following a similar rationale as in the previous studies, Study 3 also included a measure assessing general positivity. Considering that awe is conceptualized predominantly as a positive emotion (Bonner & Friedman, 2011; Keltner & Haidt, 2003; Shiota et al., 2007), its effect may depend on the general positivity of awe. To rule out this possibility, we assessed general positivity to ensure the negative association between awe and prejudice holds beyond the general positivity disposition.

### Method

#### Participants

**FIGURE 2:**
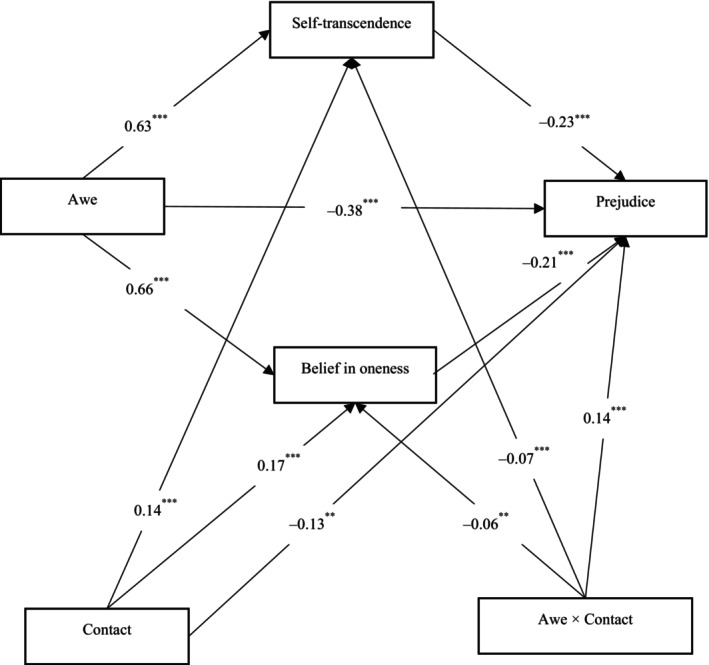
Moderated mediation model for Study 3. Standardized coefficients are displayed; **p < .01; ***p < .001.

Consistently with previous studies, we considered eligible only those participants who self‐identified as heterosexual (“Please answer anonymously in which of the following categories you self‐categorize: A. heterosexual B. LGBT C. Other”); those who did not identify as heterosexual were not considered. With the aim of securing a substantial number of participants to enhance the robustness of our results and ensure that our conclusions are well‐supported, we recruited 1287 participants to complete a paper‐and‐pencil questionnaire in exchange for a monetary reward (6 RMB). As for exclusion criteria, prior to data analysis, 62 participants were excluded for either failing an attention check or providing the same numeric response throughout the survey without variation or having missing data (i.e. less than 70% of the study completed, *N* = 37). The final sample comprised 1188 participants (611 women) aged between 18 and 52 (*M* = 30.35, *SD* = 9.27). As for previous studies, all participants included in the analyses reported being heterosexual. As before, we ran a Monte Carlo power sensitivity analysis (Schoemann et al., 2017; Thoemmes et al., 2010). The selected model was two parallel mediators, having one moderator conditioning both the independent variable → mediator pathways and the independent variable → outcome pathways, with 10,000 replications, 20,000 Monte Carlo draws per replication, 95% confidence level and 1234 as random seed. The simulation was based on the standardized coefficients emerging from the mediation model tested in Study 3 (*see* Figure 2). The power analysis indicated that with 1188 participants we had >99% statistical power to detect both moderated mediation effects (i.e. the awe × contact indirect effects via self‐transcendence and belief in oneness, respectively).

#### Procedure

Participants were recruited in Chinese public areas (e.g. public parks and squares), a sampling procedure employed in several previous studies (Panno et al., 2018; Wang et al., 2022); they were invited to take part in a research project exploring emotional experiences and social interactions. Upon obtaining consent, all participants were asked to individually complete a paper‐and‐pencil questionnaire, including a battery of measures, described below. To improve data quality, an attention check was included (“For this question, to show that you have read these instructions carefully, please select all five of the answers below”: A. Extremely important; B. Very important; C. Somewhat important; D. Only a little important; E. Not at all important). Before finishing the questionnaire, participants provided their demographic information; then, they were thanked, debriefed and rewarded for their participation.

#### Measures

Participants completed the following measures (*see* Table 1 for means, standard deviations and correlations among variables).

**TABLE 1:** Means and correlations among key variables (Study 3).

	*M*	*SD*	1	2	3	4	5
1. Prejudice	2.71	0.89	1.00				
2. Belief in oneness	2.99	1.00	−.69***	1.00			
3. Self‐transcendence	3.86	1.60	−.70***	.59***	1.00		
4. Awe	3.07	1.04	−.75***	.71***	.71***	1.00	
5. Contact	2.26	1.17	−.39***	.34***	.31***	.25	1.00

*Note*: The response scale ranges from 1 to 5 for prejudice, belief in oneness, and awe, from 1 to 7 for self‐transcendence and contact.

*** *p* < .001.

##### Awe

We assessed awe with the Awe Scale (Zhao, 2017). Participants responded to 20 items inquiring about their experience of awe (e.g. “I can often appreciate the charm of nature”), on a 5‐point scale ranging from 1 (*never or very rarely true*) to 5 (*very often or always true*). Higher scores denote stronger feelings of awe (*α* = .96).

##### Contact

Participants responded to 5 items capturing cross‐group interaction individuals had with sexual minority individuals, adapted from Voci and Hewstone (2003) (e.g. “How often do you meet sexual minority individuals in your daily life?”). Responses were provided on a 7‐point Likert scale ranging from 1 (*never*) to 7 (*usually*). Higher scores indicated greater frequency of intergroup contact experiences with members of sexual minority group (*α* = .91).

##### Self‐transcendence

Self‐transcendence was assessed in the same manner as in Study 2 (*α* = .84).

##### Belief in oneness

Belief in oneness was measured in the same manner as in Study 2 (*α* = .84).

##### Prejudice

We administered an adapted version of the attitudes towards sexual minority individuals scale (Walch et al., 2012). Participants indicated their level of agreement to 20 items (e.g. “It would be beneficial to society to recognize sexual minority as normal”) employing a 5‐point Likert scale ranging from 1 (*strongly disagree*) to 5 (*strongly agree*). In the original version of the scale, the target group was transgender people, in our adaptation we replaced the reference to transgender people with “sexual minority people”. Higher scores reflect greater prejudice towards sexual minority individuals (*α* = .93).

##### General positivity disposition

As a measure of participants' general disposition towards positive states, we employed the positive affect subscale of the positive and negative affect scale (PANAS; Watson et al., 1988). Participants answered 10 items asking how they felt during the past month (e.g. “interested”; “excited”), employing a 5‐point Likert scale ranging from 1 (*not at all*) to 5 (*very much*). Higher scores indicated a greater general positivity disposition (α = .85).

### Results and discussion

Means, standard deviations and correlations among the variables are presented in Table 1. To test our hypotheses,^3^ we performed a bootstrapped moderated mediation analysis with 10,000 resamples using SPSS PROCESS Model 8 (Hayes, 2018). To determine the statistical significance of the standardized effects, 95% confidence intervals were used. In the model, awe served as the predictor, self‐transcendence and belief in oneness as parallel mediators and prejudice as the dependent variable. We also controlled for general positivity disposition. As for contact, we tested its moderating role on the relationship between awe and each of the two mediators (i.e. self‐transcendence and belief in oneness) and on that between awe and the dependent variable (i.e. prejudice).

**FIGURE 3:**
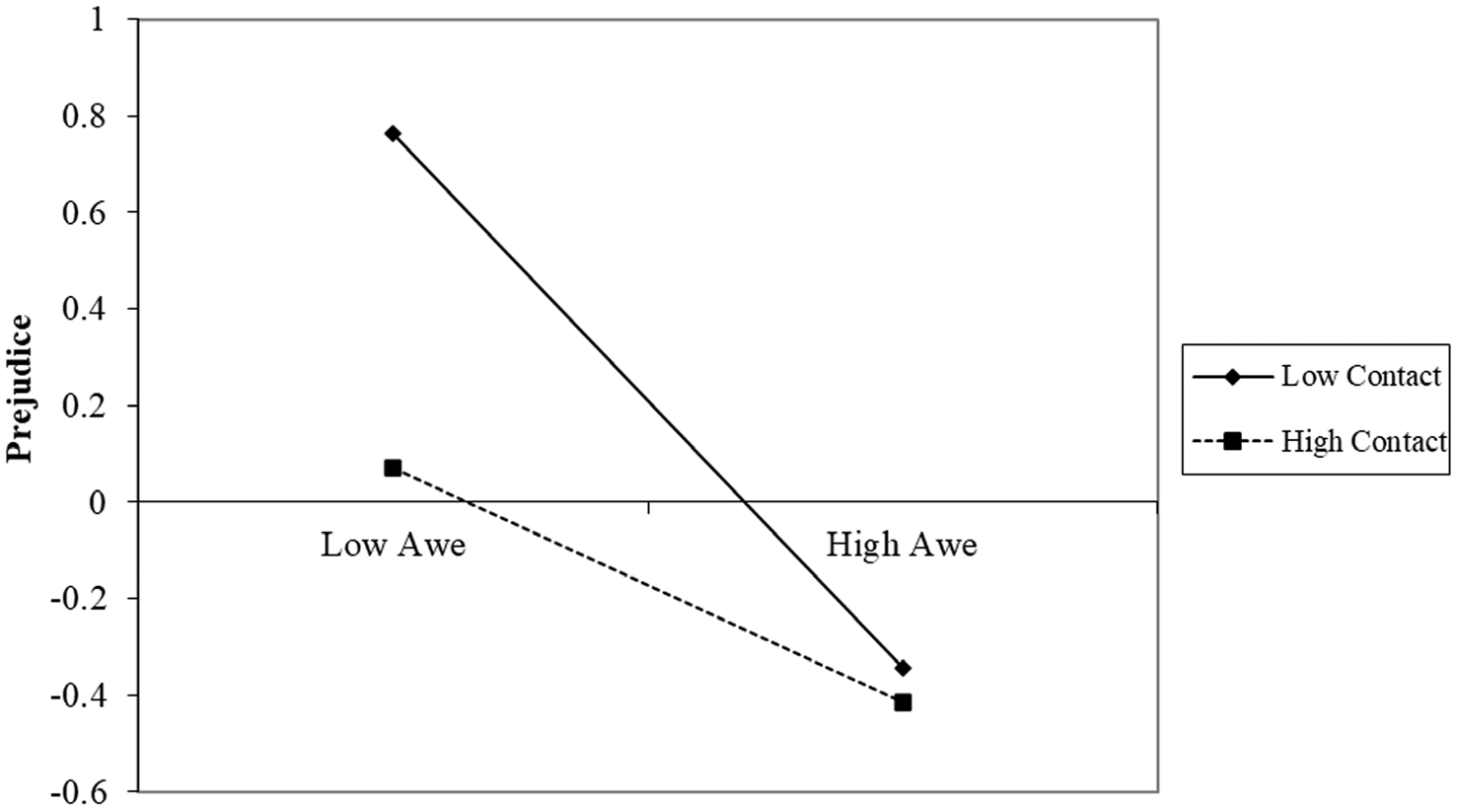
Decomposition of the interaction between awe and contact for different levels of contact (high = +1 *SD*; low = −1 *SD*), dependent variable: Prejudice (Study 3).

Results (presented in Table 2 and Figure 2) aligned with H3. As predicted, we found a significant interaction effect between awe and contact on prejudice, corroborating our hypothesis on the moderating role of contact in the relationship between awe and prejudice towards sexual minority members. We conducted a simple slope analysis to further disentangle the interaction effect of contact with awe on prejudice. In line with our prediction, results (Figure 3) showed that awe had stronger negative associations with prejudice towards sexual minority individuals among participants with less contact (−1 *SD*, *B* = −0.53, *p* < .001); awe was still significantly associated with reduced prejudice among participants with more frequent contact, though the effect was weaker (+1 *SD* above the mean, *B* = −0.20, *p* < .001).

**TABLE 2:** Regressions conducted for the moderated mediation analysis (Study 3).

Self‐transcendence	*R*	*R* ^*2*^	*F*	*p*
	.73	.53	335.23	<.001
	***β***	***SE***	***t***	***p***
Awe	.63	.03	24.83	<.001
Contact	.14	.02	6.56	<.001
Awe × Contact	−.07	.02	−3.76	<.001
**Belief in oneness**	***R***	***R*** ^**2**^	***F***	***p***
	.74	.54	352.63	<.001
	*β*	*SE*	*t*	*p*
Awe	.66	0.03	26.43	<.001
Contact	.17	0.02	8.50	<.001
Awe × Contact	−.06	0.02	−3.14	<.01
**Prejudice**	***R***	***R*** ^**2**^	***F***	***p***
	.83	.69	435.02	<.001
	***β***	***SE***	***t***	***p***
Awe	−.38	0.03	−13.15	<.001
Self‐transcendence	−.23	0.02	−9.49	<.001
Belief in oneness	−.21	0.02	−8.78	<.001
Contact	−.13	0.02	−7.53	<.001
Awe × Contact	.14	0.02	8.89	<.001
General positivity disposition	−.001	0.02	−0.06	.95

**FIGURE 4:**
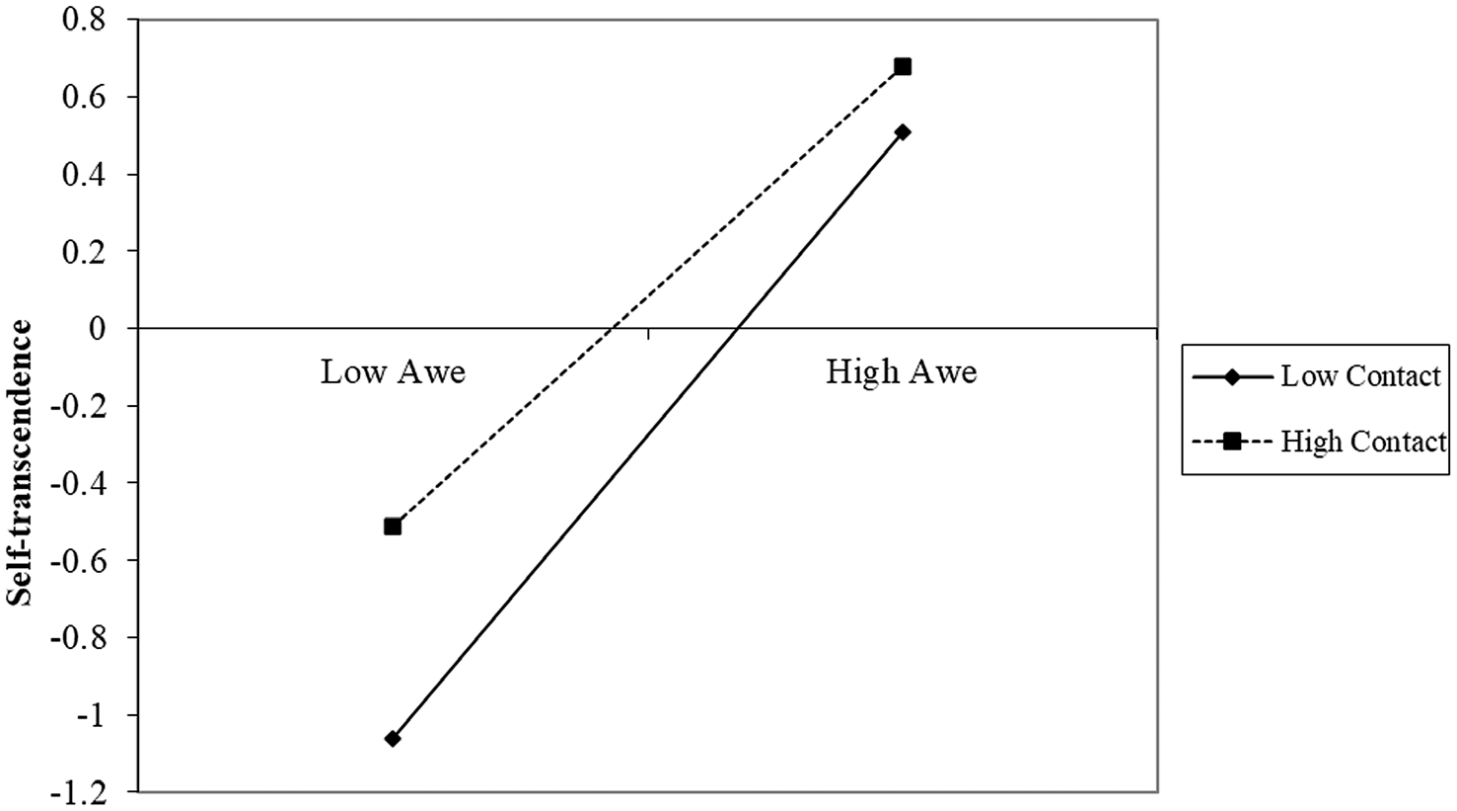
Decomposition of the interaction between awe and contact for different levels of contact (high = +1 *SD*; low = −1 *SD*), dependent variable: Self‐transcendence (Study 3).

**FIGURE 5:**
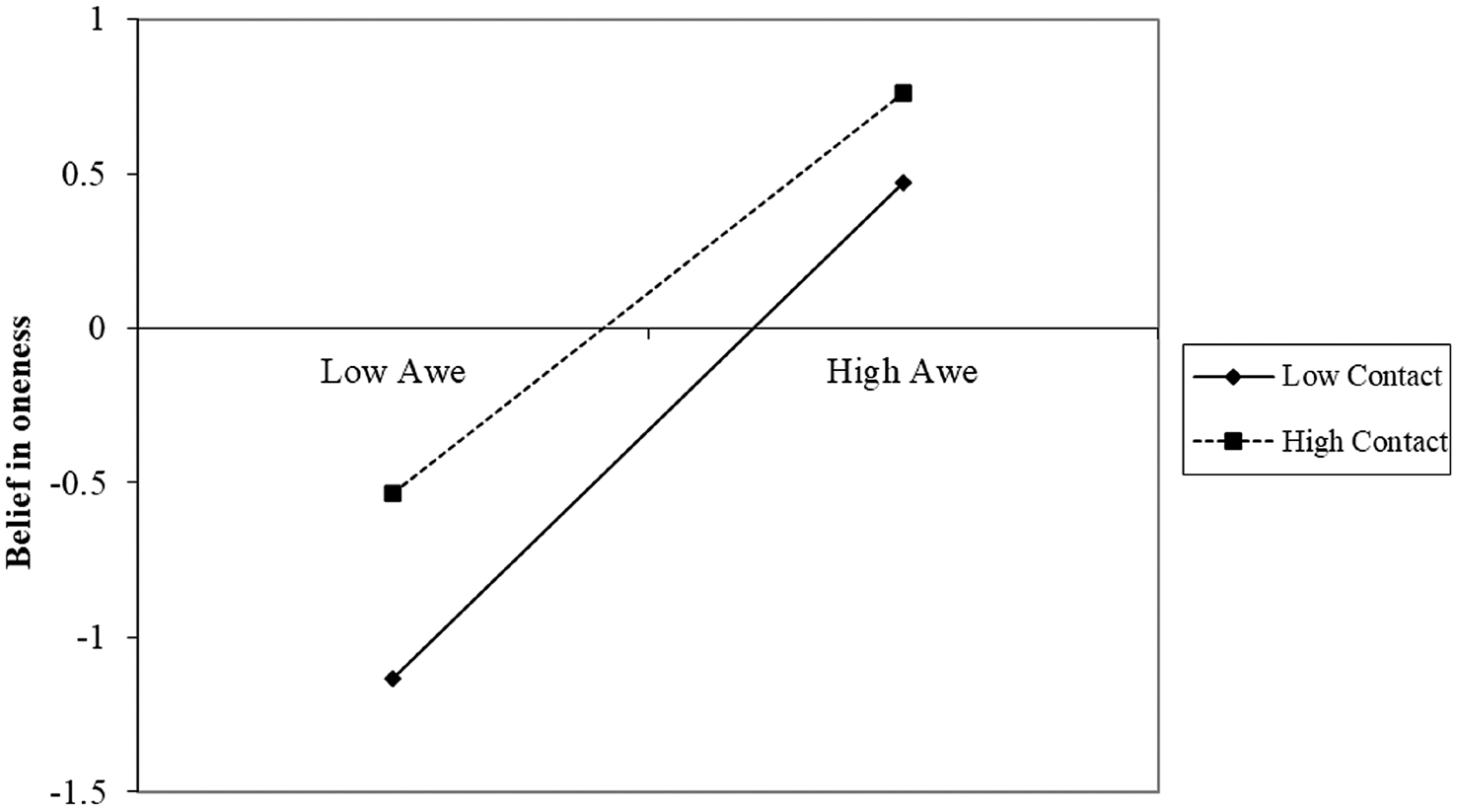
Decomposition of the interaction between awe and contact for different levels of contact (high = +1 *SD*; low = −1 *SD*), dependent variable: Belief in oneness (Study 3).

Consistently, we also found significant interaction effects between awe and contact on both self‐transcendence and belief in oneness. Again, we ran simple slope analyses to further disentangle these interaction effects. As for self‐transcendence (Figure 4), results showed that awe had a stronger association with an enhanced sense of self‐transcendence among participants with less (−1 *SD*, *B* = 0.71, *p* < .001) rather than more interactions with sexual minority individuals (+1 *SD*, *B* = 0.54, *p* < .001). The same pattern was found for belief in oneness (Figure 5): awe had a stronger association with greater belief in oneness among participants with less (−1 *SD*, *B* = 0.72, *p* < .001) rather than more interactions with sexual minority individuals (+1 *SD*, *B* = 0.58, *p* < .001).

Finally, concerning the indirect associations between awe and prejudice, not only did we replicate the pattern of associations found in Study 2, by showing that awe –also when assessed at the dispositional level– is indirectly associated with less prejudice via heightened self‐transcendence and belief in oneness; we also found that awe was indirectly associated with reduced prejudice more strongly when contact was low (self‐transcendence: *b* = −0.16, *SE* = 0.02, 95% CI [−0.21, −0.12]; belief in oneness: *b* = −0.16, *SE* = 0.03, 95% CI [−0.21, −0.10]) rather than high (self‐transcendence: *b* = −0.12, *SE* = 0.02, 95% CI [−0.17, −0.09]; belief in oneness: *b* = −0.13, *SE* = 0.02, 95% CI [−0.17, −0.08]).

Overall, Study 3 aimed to delve deeper into the relationship between awe and prejudice. Beyond taking into account the mediating role of self‐transcendence and belief in oneness—consistently with Study 2—Study 3 aimed at making a step further by testing the moderating role of contact. This could provide a preliminary answer to the question of *for whom* awe is negatively associated with prejudice more strongly. We tested this hypothesis in a large cross‐sectional study; results of a mediated moderation model supported the moderating role of contact in explaining inter‐individual variability in the relationship between awe and prejudice. More specifically, greater levels of awe were associated with lower prejudice, especially for individuals who had less contact experiences. The same pattern was found when testing the relationship of awe with self‐transcendence and belief in oneness, respectively.

## GENERAL DISCUSSION

The growing literature on awe is consistently showing how it can promote a wide range of benefits both at the individual and at the social level. These benefits include the pursuit of an authentic self (Jiang & Sedikides, 2022), sustaining or promoting health status (Bai et al., 2021), enhancing collective engagement (Bai et al., 2017) and increasing prosocial outcomes (Stamkou et al., 2023). The aim of the present research was to test whether the beneficial effects of awe also extend to the field of intergroup relations, focusing on the link between awe and prejudice, as well as the investigation of its underlying mechanisms and psychological conditions. In other words, we aimed at exploring whether, how and for whom awe is (more) negatively related to prejudice towards sexual minorities. We did that in three studies—two experimental and one cross‐sectional—involving independent samples of heterosexual participants and considering sexual minority group members as the target of prejudice.

Results largely supported our claims about the relationship between awe and prejudice, in line with preliminary research showing its beneficial effect within the context of gender‐ and race‐based stereotypes (Stell, 2018), interethnic relationships (Stamkou et al., 2023) and for other forms of stigma, such as towards obese people (Lv et al., 2024) and people with AIDS (Luo et al., 2022). Notably, this link was consistent across various manipulations, measures, samples and methodologies, highlighting its robustness.

In line with H1, Studies 1 and 2 showed that people experiencing awe (vs. a neutral state) reported lower prejudice towards sexual minority group members. Moreover, Study 2 showed that inducing awe (compared to amusement and a neutral state) resulted in lower prejudice towards sexual minority group members via two mechanisms related to changes in self and world perceptions, namely increased self‐transcendence and belief in oneness, thus supporting H2. Finally, in line with H3, contact quantity was found to have a moderating role on the relationships among the above‐mentioned variables. Specifically, awe and its mediators were particularly strongly associated with reduced prejudice among individuals having fewer intergroup encounters. The same pattern, although weaker, was observed among participants having more frequent intergroup interactions. It is worth noting that all these effects occurred independently of other (non‐self‐transcendent) emotions, underscoring the plausibility that awe's impact on prejudice toward sexual minority group members simply depends on the benefits of experiencing positive emotions. On the contrary, our results suggest that experiences of positive emotions in general and of awe are not overlapping and that the latter has a specific and unique effect on prejudice.

The present findings can be interpreted combinedly under the lens of the common ingroup identity model (Gaertner et al., 1993). The model proposes re‐categorization as a relevant prejudice reduction strategy: by conceiving themselves as member of a single overarching group, rather than two separate groups, people can embrace more positive intergroup attitudes. The fact that awe favours a shift to a more inclusive identity has already received empirical support. Experimental studies employing both nature‐ and non‐nature‐based awe manipulations showed that awe increases common ingroup identity (Lv et al., 2024) and favours global identification processes (Seo et al., 2023). Additionally, our studies found that changes in self and world perceptions (i.e. self‐transcendence and belief in oneness) following experiences of awe partially explain the relationship between awe and reduced prejudice. We argue that these perceptual changes could further facilitate the shift towards a more inclusive identity, thereby reinforcing prejudice reduction. Consistently, awe and its underlying mechanisms share their properties with intergroup contact, which has been widely demonstrated as an effective strategy for changing perceptions of group boundaries and favouring a shift from an “us vs. them” to a more inclusive “we” perspective. This is why intergroup contact and awe might be seen as complementary tools, with the latter being particularly useful when contact is difficult to achieve.

Importantly, we argue that these novel findings might be particularly relevant in contexts where stigma towards individuals belonging to sexual minorities still exists, such as the one where our data were collected. In such contexts, contact opportunities are often scarce, as suggested by estimates reporting that less than 10% of sexual minority group people publicly disclosed their sexual orientation, gender identity and gender expression (United Nations Development Programme, 2016). Therefore, individuals belonging to the sexual minority group are often “invisible minorities” due to stigma and a lack of opportunities for disclosure to the larger majority population. In this context, awe‐induced identity shifts can provide alternative pathways to achieve prejudice reduction, greater acceptance and understanding.

The present investigation has several implications that span both the field of intergroup relations and the realm of awe research. On one side, our results contribute to the study of prejudice by empirically establishing a negative relation between awe and prejudice towards sexual minority groups, therefore identifying a new deterrent of intergroup prejudice. On the other side, the present investigation also has implications for the science of awe, by broadening knowledge on its range of effects. Indeed, the present research is the first, to our knowledge, to systematically explore awe in relation to prejudice, suggesting that awe has important implications that extend beyond the individual (Bai et al., 2017) and the interpersonal (Piff et al., 2015) levels. Moreover, the exploration into the underlying psychological processes and moderators of the awe‐prejudice link provides—to our knowledge, for the first time—important insights into the intricacy and complexity of their relation.

In this sense, our studies could serve as a step forward in the development of interventions that apply constructs related to positive psychology, including self‐transcendence emotions, to the reduction of prejudice, an area of research that is still at its early stages (for a review, *see* Sanders et al., 2023). In particular, our results on the role of intergroup contact in the relationship between awe and prejudice have relevant practical implications.

As previously anticipated, awe can serve as an alternative to contact, when difficult to achieve, while still reducing prejudice even among those with frequent intergroup encounters, suggesting that contact does not prevent the effect of awe. In other words, our results further corroborate the fact that awe and contact are not competing but complementary strategies, which can be combined to promote (even more) positive outcomes in terms of prejudice reduction. This finding is particularly encouraging, given the extensive body of socio‐psychological literature identifying intergroup contact as a highly effective tool for reducing prejudice (Dovidio et al., 2017; Pettigrew & Tropp, 2013; Vezzali & Stathi, 2020). Upon these premises, we argue that our results can illuminate and guide policymakers who plan interventions aimed at reducing prejudice in at least two ways. First, by helping them to detect the target population that would benefit most from an awe intervention to reduce prejudice, namely those who have few contact experiences with members of an outgroup: as discussed in the previous paragraph, this could be particularly useful for “invisible” minorities, or under‐represented minority groups with whom possibilities of contact are often scarce. Second, our results could help maximize the effectiveness of inducing awe, by combining it with more frequent intergroup encounters. In this sense, as a future development, we plan to design and test an Awe‐Intergroup Contact (AIC) programme, where empirically supported awe‐inducing techniques and contact guidance are combined organically and implemented step by step. This programme might have the potential to favor people's experiences of awe, as well as to have high‐quality interactions with members of an outgroup.

## LIMITATIONS AND FUTURE DIRECTIONS

Though the current research yielded novel and promising results, some limitations should be acknowledged in order to envisage key future directions. First, due to limited resources, all studies relied on samples from a single country; thus, our results might not be generalizable to other cultural contexts (Simons et al., 2017). We do indeed acknowledge the rich cultural variation in human emotions (for recent debates on this topic see Lindquist et al., 2022 but also Mesquita, 2022). As for awe specifically, research on dispositional awe found that participants from the US, Iran, Malaysia and Poland report significant differences in the frequency in which they experience awe, with US participants displaying the highest levels (Razavi et al., 2016). Moreover, if in the Western cultural context (US) awe is mainly perceived as a positive emotion, in some Eastern Asian contexts (Japan) it has been shown to be a mixed emotion (Nakayama et al., 2020). Given the complexity of awe's conceptualization across cultures, with variations also in the degree people experience it, future studies should investigate the effect of awe on prejudice in other cultural contexts to enhance the generalizability of our findings. Despite this limitation, it is worth noting that the manipulations employed in our studies were found to be effective in both Eastern (Jiang & Sedikides, 2022) and Western (Hornsey et al., 2018; Piff et al., 2015) contexts.

Second, we employed short videos to induce feelings of awe, measuring prejudice right after the manipulation. Arguably, to properly uncover the lasting effect of awe upon prejudice, future studies should test its effects on prejudice over a longer period of time. Importantly, while this approach aimed to ensure conceptual clarity and is similar to previous research (Bai et al., 2017), we also acknowledge the risk of potential demand effects due to the definition of awe provided to participants in Study 1 and 2.

Third, when assessing prejudice towards the sexual minority group, we treated it as a whole, without taking into account specific subgroups within the sexual minority community. This is particularly true for Studies 1 and 2, where we administered Wright et al.'s (1997) semantic differential scale, commonly employed to assess attitudes towards a wide variety of groups and therefore not specifically tailored to the outgroup of sexual minority individuals. Despite in Study 3 we employed a scale designed to assess attitudes towards the sexual minority group (which replicated results obtained in the first two studies), it is worth noting that we adapted the scale, by replacing the target group, which originally was transgender people, with the broader reference to “sexual minority people.” We must acknowledge that we might have oversimplified the complexity and nuances of sexual prejudice, by not explicitly taking into account subgroups belonging to this minority. Future studies could possibly replicate our findings by using validated measures that account for the diversity within the sexual minority community. Somehow relatedly, it might also be important to extend these findings to other disadvantaged groups, such as people of different ethnicities or with disabilities, and to explore the effects of awe beyond prejudice reduction, investigating whether inducing feelings of awe leads to other positive outcomes at the intergroup level, such as increased collective action in support of minorities.

Fourth, in the present investigation, we did not differentiate between positive and negative feelings of awe. It is worth noting that stimuli eliciting awe can also include nationalist propaganda, rousing speeches, or large rallies. The experience of such stimuli can be accompanied by negative emotional states, such as anxiety or fear (Keltner & Haidt, 2003) and might not favour common identification processes, rather strengthening an “us vs. them” mentality reinforcing ingroup bias and increasing prejudice. In this sense, emerging research provides mixed findings. On the one hand, it was documented that positive and negative experiences of awe entail different underlying mechanisms, subjective experiences and physiological correlates (Gordon et al., 2017). On the other hand, some studies suggest that both positive and negative awe, evoked by threatening experiences, are associated with increased prosocial values and behaviours (Piff et al., 2015; Seo et al., 2023). However, it is also worth noting that these studies employed nature‐based negative awe, therefore the question of whether human‐based stimuli eliciting negative awe could lead to negative interpersonal and intergroup outcomes remains still open. Understanding whether and how these stimuli differently affect intergroup outcomes could help in designing effective awe‐based interventions that maximize its benefits while lessening or avoiding its possible negative downsides.

Fifth, following extant awe research routine (Jiang & Sedikides, 2022; Piff et al., 2015), in our studies we assessed and controlled for six primary emotions to ensure that the effect of awe would hold when controlling for a range of primary emotional states. However, we did not measure other self‐transcendence emotions; therefore, we do not have a direct test of whether the experimental manipulation specifically elicits awe and no other emotions of the same taxonomy. Future research should possibly fill this gap, especially when employing awe manipulations that are based on human‐based stimuli, rather than nature‐based stimuli (Stellar et al., 2017).

Sixth, it is worth noting that in Study 3 we used a measure of contact quantity, since we predicted that the effects of awe would depend on the (lack of) occurrence of participants' contact experiences. It should however be noted that contact effects are generally stronger for quality than quantity of contact (Hodson & Hewstone, 2013; but see Vezzali et al., 2023). More specifically, the valence of the contact experience may drive the effects of awe, such that positive contact experiences can boost prejudice reduction, while negative contact experiences can increase prejudice (for a review on negative contact, see Schafer et al., 2021). We believe future studies can include contact valence in the study of awe to better understand when it will have positive or negative effects on prejudice.

Finally, two additional limitations are specific to Study 3. The first limitation lies in the correlational nature of Study 3. We acknowledge the lack of causality in our data; to strengthen the case for moderated mediation analysis, future researchers should replicate this result by providing experimental evidence supporting the causal relationships among the study variables. Second, another important point to acknowledge in this section concerns the clustered nature of the data of Study 3, which revealed two subgroups of participants (see Endnote 3 for a reference to that and the Supporting Information for detailed analyses). One cluster was characterized by relatively low levels of awe, contact, self‐transcendence and belief in oneness, alongside relatively high levels of prejudice. The other cluster displayed the opposite pattern, scoring relatively high on awe, contact, self‐transcendence and belief in oneness, and relatively low on prejudice. We can only hypothesize about the factors driving the emergence of these clusters, taking into account a series of variables that are related to prejudice and that are (or could be) relevant also to awe. Extensive research suggests for instance that people with high prejudice tend to exhibit traits such as high Social Dominance Orientation (SDO), high Right‐Wing Authoritarianism (RWA) and a strong need for cognitive closure (Hodson & Dhont, 2015). SDO and RWA entail a competitive worldview characterized by the need to preserve inequalities and social hierarchies, and conservatism and rigidity towards authority, respectively. These characteristics somehow “clash” with those of awe and its underlying mechanisms, which entail, as we argue in this work, a perceptive change of group boundaries, favouring a more inclusive (rather than competitive) worldview. These speculations are consistent with previous literature showing as an example that awe favours global identification processes (Seo et al., 2023) and increases common ingroup identity (Lv et al., 2024)—which is typically negatively associated with prejudice (Dovidio & Gaertner, 1999)—and that is negatively associated with cognitive closure (Shiota et al., 2007). Our results represent a meaningful contribution to the literature. Specifically, the identification of a subgroup with low prejudice and high levels of awe and its underlying mechanisms can be a structurally relevant indicator underscoring the potential of awe to reduce not only prejudice but also to impact more extensively on a set of dispositions and beliefs broadly related to greater openness towards diversity. Nevertheless, we acknowledge the need for a direct test of whether these dispositional variables drive the emergence of these two profiles. Future studies should address this gap to elucidate the factors underlying these clusters.

In conclusion, and despite these limitations, the present research represents a first effort that sheds light on the complex connection between awe and prejudice, revealing awe as a potent force in reducing bias against sexual minority group members.

## AUTHOR CONTRIBUTIONS

**Wang Changcheng:** Conceptualization; investigation; methodology; data curation; formal analysis; funding acquisition; writing – original draft; writing – review and editing. **Alice Lucarini:** Data curation; writing – original draft; writing – review and editing; formal analysis. **Veronica Margherita Cocco:** Data curation; writing – original draft; writing – review and editing. **Kim Dierckx:** Formal analysis; writing – review and editing. **Loris Vezzali:** Data curation; writing – original draft; writing – review and editing; supervision.

## FUNDING INFORMATION

This paper was funded by the National Social Science Fund of China (23CSH048), General Project of Philosophy and Social Science Research in Colleges and Universities in Jiangsu Province (TJZ223021) and Talent Introduction Research Start‐up Fund of Nanjing University of Posts and Telecommunications (NYY223011).

## CONFLICT OF INTEREST STATEMENT

The authors declare no conflicts of interest.

## INFORMED CONSENT

The authors declare that informed consent was obtained.

## Supporting information


Appendix S1.

## Data Availability

The data that support the findings of this study are available in OSF: https://osf.io/guwp3/?view_only=c7880ee430eb4c418a217f346e6d13d5.

## Footnotes

^1^
Importantly, for both Study 1 and Study 2, we also performed a one‐way ANOVA to test whether the awe‐eliciting (vs. control) video had effects on the other emotions used as fillers. Results showed that these emotions did not differ significantly between conditions (see Tables S1 and S2).

^2^
See the Supporting Information for tests of alternative models (and model comparisons) with self‐transcendence and belief in oneness as sequential mediators.

^3^
It should be noted that a latent profile analysis (LPA; reported in detail in the Supporting Information) indicated the presence of two subgroups in the data, one (*N* = 542) scoring low on awe, contact, self‐transcendence and belief in oneness, and high on prejudice; and another (*N* = 646) displaying the reverse pattern. However, given that the results were virtually identical across subgroups, we decided, for the sake of brevity, to collapse these groups and report the moderated mediation analysis for the full sample only.
