# Supplementary material for: Building bridges with awe: Exploring underlying mechanisms and moderators of the relationship between awe and prejudice towards sexual minority group members
Source: Br J Soc Psychol. 2025 Apr 2;64(2):e12884. doi: 10.1111/bjso.12884 (PMC11963834; doi:10.1111/bjso.12884)
Supplement: Supplementary file 1 — Appendix S1. [file BJSO-64-0-s001.docx]

**Building Bridges with Awe: Exploring Underlying Mechanisms and Moderators of the Relationship between Awe and Prejudice toward Sexual Minority Group Members**

**Supplementary Materials**

# Comments on the power analyses of Studies 1 and 2

**Study 1**

As stated in the manuscript, in Study 1, we ran a sensitivity power analysis for a one-way between-subjects design with two conditions, employing the software G*Power (Faul et al., 2007). Results revealed that with *α* = 0.05 and 1-*β* = 0.80, the minimum detectable effect size was *f* = 0.14, which can be considered a small effect (Cohen, 1988). Importantly, the sensitivity power analysis confirmed that our study was well-designed to detect even small effects (*f* = 0.14) with 80% power. Results of the one-way ANOVA testing the effect of the awe-inducing condition (vs. control) on prejudice toward LGBT individuals (H1) showed a significantly different degree of prejudice depending on the experimental condition, *F*(1, 403) = 132.03, *p* < 0.001, η^2^_p_ = 0.24 (corresponding to *f* ≈ 0.56). The effect size observed in our study is larger than the minimum detectable effect, indicating that we detected a robust and significant effect.

**Study 2**

As stated in the manuscript, in Study 2, we ran a sensitivity power analysis for a one-way between-subjects design with three conditions, employing the software G*Power (Faul et al., 2007). Results revealed that with *α* = 0.05 and 1-*β* = 0.80, the minimum detectable effect size was *f* = 0.14, which can be considered a small effect (Cohen, 1988). Importantly, the sensitivity power analysis confirmed that our study was well-designed to detect even small effects (*f* = 0.14) with 80% power. Results of the one-way ANOVA testing the effect of the awe-inducing condition (vs. amusement vs. control) on prejudice toward LGBT individuals (H1) showed a significantly different degree of prejudice depending on the experimental condition, *F*(2, 506) = 68.91, *p* < .001, η^2^_p_ = 0.21 (corresponding to *f* ≈ 0.52). The effect size observed in our study is larger than the minimum detectable effect, indicating that we detected a robust and significant effect.

# Additional ANOVAs on Filler Emotions

# Study 1

As for Study 1, results of a one-way ANOVA to test whether the awe-eliciting (vs. control) video had effects on the other emotions used as fillers showed that these emotions did not differ significantly between conditions (Table S1).

**Table S1. Means, Standard Deviations, and ANOVA results for filler emotions in Study 1**

|  | Anger | |  | Pride | |  | Disgust | |  | Fear | |  | Happiness | |  | Sadness | |
| --- | --- | --- | --- | --- | --- | --- | --- | --- | --- | --- | --- | --- | --- | --- | --- | --- | --- |
|  | *M* | *SD* |  | *M* | *SD* |  | *M* | *SD* |  | *M* | *SD* |  | *M* | *SD* |  | *M* | *SD* |
| Awe | 1.13^a^ | 0.39 |  | 1.25^a^ | 0.61 |  | 1.12^a^ | 0.36 |  | 1.31^a^ | 0.58 |  | 1.29^a^ | 0.58 |  | 1.13^a^ | 0.36 |
| Control | 1.20^a^ | 0.49 |  | 1.21^a^ | 0.51 |  | 1.17^a^ | 0.40 |  | 1.35^a^ | 0.73 |  | 1.29^a^ | 0.61 |  | 1.17^a^ | 0.41 |
|  | *F*(1, 409) = 2.27, *p* = 0.13 | |  | *F*(1, 409) = 0.66, *p* = 0.42 | |  | *F*(1, 409) = 2.16, *p* = 0.14 | |  | *F*(1,409) = 0.36, *p* = 0.55 | |  | *F*(1, 409) = 0.01, *p* = 0.94 | |  | *F*(1, 409) = 1.16, *p* = 0.28 | |

**Study 2**

Consistent with Study 1, in Study 2 we performed one-way ANOVAs to test the effect of the awe-eliciting video (vs. amusement and control) on the emotions used as fillers. Results showed that these emotions did not differ significantly between conditions (see Table S2 in the Supplementary Materials). Post-hoc Bonferroni-corrected comparisons showed that there was no significant difference among the three conditions in the degree to which they elicited the filler emotions (p ≥ 0.35). Nevertheless, we found a tendency for Happiness to be higher in the amusement (M = 1.24, SD = 0.47) condition, compared to the awe (M = 1.14, SD = 0.38) and control (M = 1.16, SD = 0.48) condition. Despite the fact that these comparisons did not reach the standard threshold of significance, this trend is in line with the results found for amusement, supporting the idea that the amusement-inducing video should increase positive emotions to a greater extent compared to the awe-inducing (and the control) condition(s) (Table S2).

**Table S2. Means, Standard Deviations, and ANOVA results filler emotions in Study 2**

|  | Anger | |  | Pride | |  | Disgust | |  | Fear | |  | Happiness | |  | Sadness | | |
| --- | --- | --- | --- | --- | --- | --- | --- | --- | --- | --- | --- | --- | --- | --- | --- | --- | --- | --- |
|  | *M* | *SD* |  | *M* | *SD* |  | *M* | *SD* |  | *M* | *SD* |  | *M* | *SD* |  | *M* | *SD* |  |
| Awe | 1.03^a^ | 0.21 |  | 1.10^a^ | 0.32 |  | 1.01^a^ | 0.11 |  | 1.08^a^ | 0.27 |  | 1.14^a^ | 0.38 |  | 1.04^a^ | 0.20 |  |
| Amusement | 1.01^a^ | 0.11 |  | 1.06^a^ | 0.29 |  | 1.01^a^ | 0.08 |  | 1.04^a^ | 0.24 |  | 1.24^a^ | 0.47 |  | 1.01^a^ | 0.11 |  |
| Control | 1.02^a^ | 0.13 |  | 1.08^a^ | 0.27 |  | 1.02^a^ | 0.15 |  | 1.03^a^ | 0.21 |  | 1.16^a^ | 0.48 |  | 1.04^a^ | 0.20 |  |
|  | *F*(2, 511) = 1.00, *p* = 0.37 | |  | *F*(2, 511) = 0.61 *p* = 0.54 | |  | *F*(2, 511) = 1.00, *p* = 0.37 | |  | *F*(2, 511) = 1.62, *p* = 0.20 | |  | *F*(2, 511) = 2.16, *p* = 0.12 | |  | *F*(2, 511) = 1.58, *p* = 0.21 | | |

## **Alternative models**

## **Study 2**

As mentioned in the manuscript, the relationship between awe and self-transcendence received wide empirical support; conversely, there is a paucity of research testing the effect of awe on belief in oneness. Similarly, the relationship between the two mediators has yet to be clearly disentangled. This is consistent with literature arguing that believing in oneness can both precede and result from perceiving one’s identity in an allo-inclusive manner (Diebels & Leary, 2019). Indeed if, on the one hand, greater beliefs that everything is part of a whole may depend on an expansion beyond one’s self-boundaries; on the other hand, believing in oneness may also affect the way people think about themselves, thus leading them to greater self-transcendence. Alternatively, these processes could likely act on two different, yet parallel, channels, with awe promoting a change in self-perceptions on the one side, and a change in worldviews and beliefs, on the other side. In the manuscript, we present this latter option, by testing a parallel mediation of self-transcendence and belief in oneness in the relationship between awe and prejudice. Here in the Supplementary Materials we report two alternative models testing the sequential path from self-transcendence to belief (Model S1) in oneness and vice-versa (Model S2). We compared these models to the one presented in the manuscript (henceforth Model 1) by computing AIC and Akaike weights (Wagenmakers & Farrell, 2004).

For both Models S1 (self-transcendence🡪belief in oneness) and S2 (belief in oneness🡪 self-transcendence), we performed bootstrapped serial mediation analyses with 10,000 resamples using SPSS PROCESS Model 6 (Hayes, 2018; Preacher & Hayes, 2004, 2008); 95% confidence intervals were used to determine statistical significance of the standardized effects.

Figure S1 illustrates Model S1 and provides path coefficients of the associations among the variables. As for indirect effects, we found support for a sequential path where the awe condition (in contrast to the amusement and control conditions; coded as awe = 2, amusement = –1, control = –1) predicted less prejudice via heightened self-transcendence, which in turn was positively associated with greater belief in oneness; the indirect effect was significant, *b* = –.04, *SE* = .01, CI [–.06, –.02].

Figure S2 illustrates Model S2 and provides path coefficients of the associations among the variables. As for indirect effects, we found support for a sequential path where the awe condition (in contrast to the amusement and control conditions; coded as awe = 2, amusement = –1, control = –1) predicted less prejudice via greater belief in oneness, which in turn was positively associated with heightened self-transcendence; the indirect effect was significant, *b* = –.03, *SE* = .01, CI [–.05, –.02].

Following, we computed AIC indexes of Model 1, Model S1, and Model S2, from which we calculate the Akaike weights (Wagenmakers & Farrell, 2004). As shown in Table S3, the Akaike weights indicated a preference for our Model 1 that, compared to the alternative models, had the highest weight, suggesting that it is the best model among those considered for explaining the data with least information loss.

Importantly, this result is consistent with the fact that parallel mediations do not require the specification of a direct causal order among mediators, which can be difficult to justify and correctly specify. Not assuming a specific causal order among mediators, parallel mediations can provide more flexibility and robustness in situations where the causal relationships among the mediators are not well understood. We acknowledge this is likely to be the case of the relationship between self-transcendence and belief in oneness. Moreover, as indicated by the data, these processes likely act on two different, yet parallel, channels, with awe promoting a change in self-perceptions on the one side, and a change in worldviews and beliefs, on the other side.

**Figure S1.** Model S1.

–0.24^***^

0.19^***^

Belief in oneness

Awe

Prejudice

Self-transcendence

–0.34^***^

–0.16^***^

0.44^***^

0.25^***^

*Note:* Standardized coefficients are displayed; ^***^*p* < .001.

**Figure S2.** Model S2.

–0.34^***^

0.11^***^

Self-transcendence

Awe

Prejudice

Belief in oneness

–0.24^***^

–0.16^***^

0.47^***^

0.30^***^

*Note:* Standardized coefficients are displayed; ^***^*p* < .001.

**Table S3. AIC, and Akaike weights for Model S1, Model S2, and Model 1**

| **Model** | **AIC** | ***w_i_* (AIC)** |
| --- | --- | --- |
| Model S1 | 3065.86 | 0,00 |
| Model S2 | 3071.94 | 0,00 |
| Model 1 | 180.88 | 1,00 |

## **Additional latent profile and moderated mediation analyses**

## **Study 3**

A closer inspection of the distribution of our focal variables revealed that the awe scale was bimodal, whereas the prejudice scale was shown to be somewhat skewed (see Figure S3 below). In addition, when checking the assumptions for our three main regression models (awe x contact on self-transcendence, awe x contact on belief in oneness, awe x contact on prejudice), the model-specific residual QQ plots signaled substantial deviations from normality (see Figure S4 below), an observation which was corroborated by the results of all four formal tests for detecting violation of the normality assumption (all *p*s < .015) – see Table S4.

**Figure S3.** Distribution of Study 3 awe scale (top panel) and Study 3 prejudice scale (bottom panel).


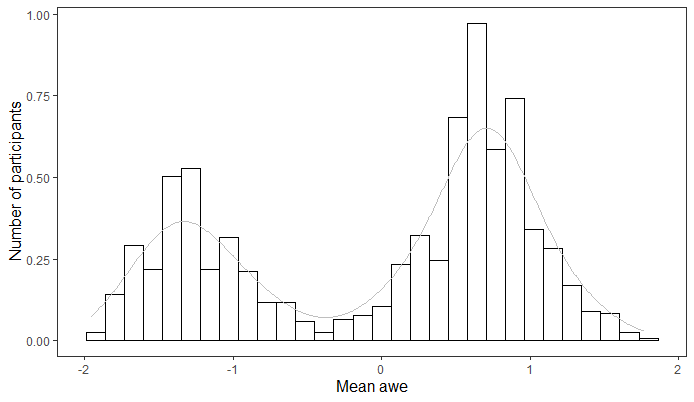

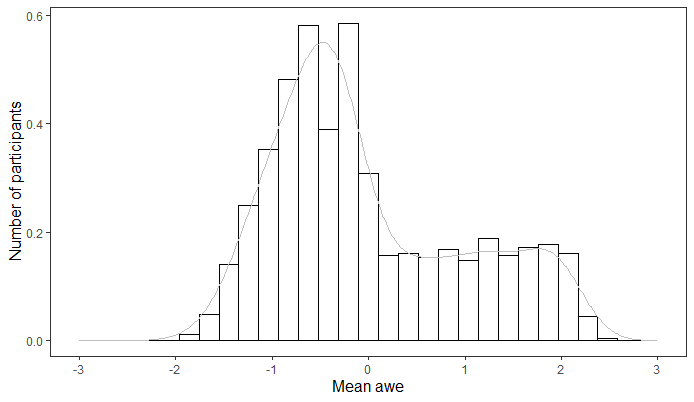


**Table S4. Results of four tests (Shapiro-Wilk, Kolmogorov-Smirnov, Cramer- von Mises, Anderson-Darling) for detecting violation of the residual normality assumption for the three main regression models.**

| Model | Test | Statistic | *p*-value |
| --- | --- | --- | --- |
| DV = Self-transcendence | Shapiro-Wilk | 0.94 | <.001 |
|  | Kolmogorov-Smirnov | 0.10 | <.001 |
|  | Cramer-von Mises | 128.76 | <.001 |
|  | Anderson-Darling | 18.27 | <.001 |
| DV = Belief in oneness | Shapiro-Wilk | 0.95 | <.001 |
|  | Kolmogorov-Smirnov | 0.07 | <.001 |
|  | Cramer-von Mises | 134.06 | <.001 |
|  | Anderson-Darling | 15.45 | <.001 |
| DV = Prejudice | Shapiro-Wilk | 0.97 | <.001 |
|  | Kolmogorov-Smirnov | 0.05 | .014 |
|  | Cramer-von Mises | 133.24 | <.001 |
|  | Anderson-Darling | 5.63 | <.001 |

**Figure S4.** Residual QQ plots for the main regression models; DVs = self-transcendence (top panel), belief in oneness (middle panel), and prejudice (bottom panel).


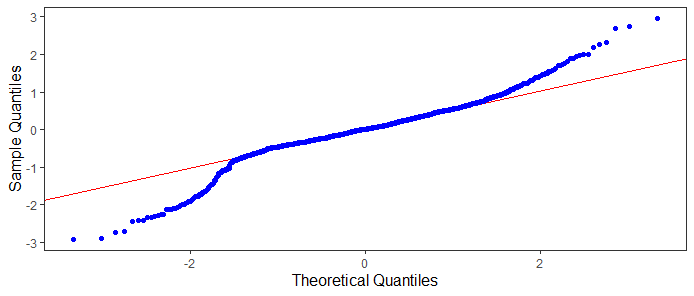

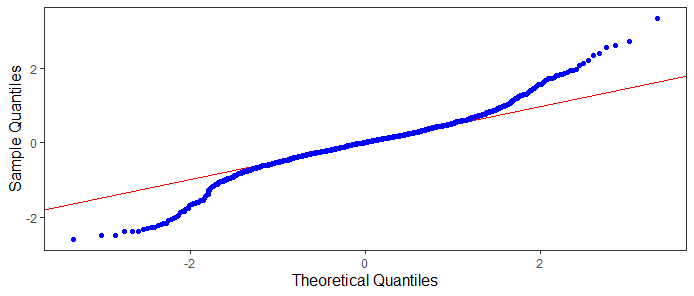

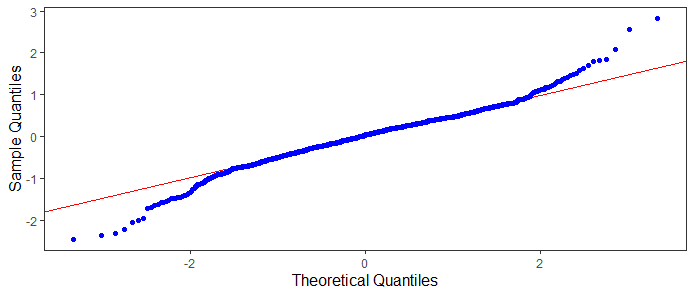


As such, to account for these deviations from normality (and violated assumptions), we decided to investigate whether different groups could indeed be identified in the data, and, if so, examine whether the results of our main moderated mediation analyses, reported in the manuscript, would hold for all emerging subgroups.

Corroborating our visual inspections of Figure S3, the results of a latent profile analysis (LPA), using the *mclust* package (Scrucca et al., 2016) in R (R Core Team, 2024), revealed the presence of two latent subgroups. A first subgroup (i.e., Latent Profile [LP] 1, *N* = 542) scored (relatively) low on awe, contact, self-transcendence, and belief in oneness, and (relatively) high on prejudice. By contrast, a second subgroup (i.e., Latent Profile [LP] 2, *N* = 646) displayed the opposite pattern, scoring (relatively) high on awe, contact, self-transcendence, and belief in oneness, and (relatively) low on prejudice. See Table S5 for an in-depth description of the subgroups in terms of the focal variables, and tests of between-group differences in these constructs.

**Table S4. Subgroup-specific means and *SD*s (between brackets), and *p*-values for Welch ANOVAs testing whether the differences in means between subgroups are significant.**

| Variable | LP 1 | LP 2 | *p*-value |
| --- | --- | --- | --- |
| Awe | 2.23 *(0.94)* | 3.78 *(0.36)* | <.001 |
| Contact | 2.03 *(1.23)* | 2.46 *(1.07)* | <.001 |
| Self-transcendence | 2.54 *(1.32)* | 4.97 *(0.72)* | <.001 |
| Belief in oneness | 2.23 *(0.96)* | 3.63 *(0.42)* | <.001 |
| Prejudice | 3.29 *(0.96)* | 2.23 *(0.41)* | <.001 |

*Notes.* LP1 = Latent Profile 1, *N* =542. LP 2 = Latent Profile 2; *N* = 646.

Next, we ran the main moderated mediation model, reported in the manuscript, for each subgroup separately. The results of these analyses are reported in detail below, see Tables S5-S6, and Figures S5-S12.

Interestingly, however, it was revealed that the main results were virtually identical across subgroups – with the sheer exception of the mediation effect of awe on prejudice through belief in oneness in LP1, and the moderated awe x contact mediation effect on prejudice through belief in oneness in the same subgroup, *p*s > .149. Given the similarity in effects between the subgroups, we thus decided to collapse LP1 and LP2 and only report the moderated mediation analysis for the full sample in the manuscript.

**Table S5**. Regressions conducted for the moderated mediation analysis—LP1 (Study 3).

| *Self-transcendence* | *R* | *R^2^* | *F* | *p* |
| --- | --- | --- | --- | --- |
|  | 0.40 | 0.17 | 26.72 | <0.001 |
|  | *β* | *SE* | *t* | *p* |
| Awe | 0.26 | 0.04 | 6.19 | <0.001 |
| Contact | 0.17 | 0.03 | 5.43 | <0.001 |
| Awe × Contact | −0.09 | 0.04 | −2.42 | <0.05 |
| *Belief in oneness* | *R* | *R^2^* | *F* | *p* |
|  | 0.48 | 0.23 | 40.77 | <0.001 |
|  | *β* | *SE* | *t* | *p* |
| Awe | 0.44 | 0.05 | 9.39 | <0.001 |
| Contact | 0.18 | 0.03 | 5.25 | <0.001 |
| Awe × Contact | −0.09 | 0.04 | −2.23 | <0.05 |
| *Prejudice* | *R* | *R^2^* | *F* | *p* |
|  | 0.80 | 0.63 | 153.35 | <0.001 |
|  | *β* | *SE* | *t* | *p* |
| Awe | −0.44 | 0.04 | −10.35 | <0.001 |
| Self-transcendence | −0.39 | 0.04 | −10.08 | <0.001 |
| Belief in oneness | −0.36 | 0.04 | −10.24 | <0.001 |
| Contact | −0.21 | 0.03 | −7.18 | <0.001 |
| Awe × Contact | 0.07 | 0.03 | 2.27 | <0.05 |
| General positivity disposition | −0.03 | 0.03 | −0.83 | 0.41 |

**Figure S5.** Moderated mediation model for LP1 in Study 3.

Belief in oneness

Awe

Prejudice

Self-transcendence

–0.39^***^

–0.44^***^

0.26^***^

Awe × Contact

–0.36^***^

0.44^***^

–0.09^*^

0.07^*^

Contact

–0.21^***^

0.17^***^

0.18^***^

–0.09^*^

*Note.* Standardized coefficients are displayed for for the moderated mediation analysis-LP1; ^***^*p* < .001, ^*^*p* < .05.

**Figure S6.** Decomposition of the interaction between awe and contact for different levels of contact (high = +1 *SD*; low = -1 *SD*), dependent variable: Self-transcendence (Study 3, LP1).

**
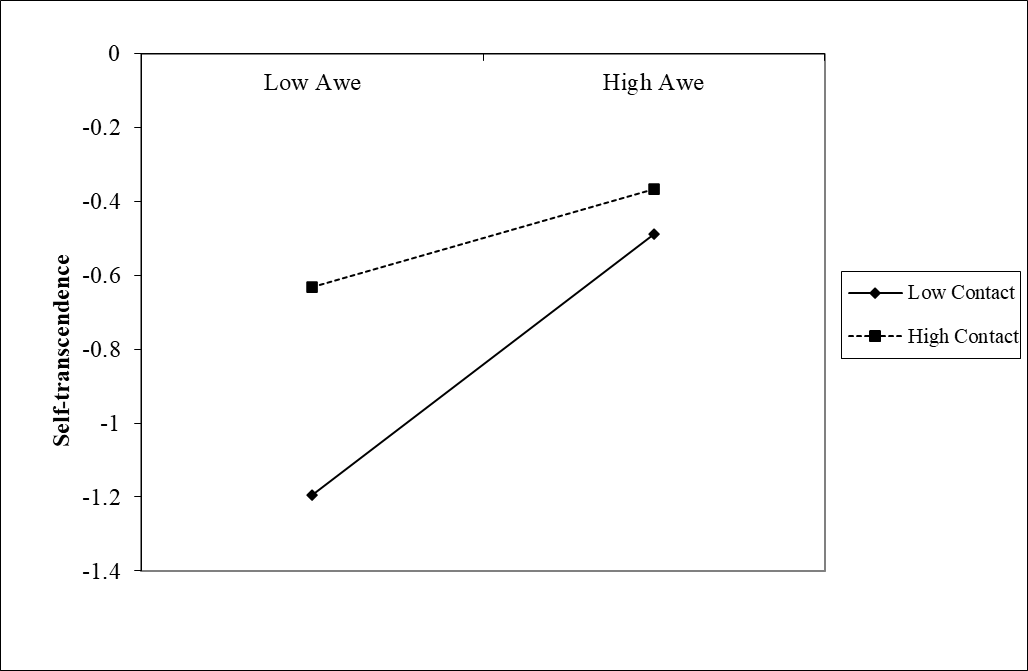
**

**Figure S7.** Decomposition of the interaction between awe and contact for different levels of contact (high = +1 *SD*; low = -1 *SD*), dependent variable: Belief in oneness (Study 3, LP1).


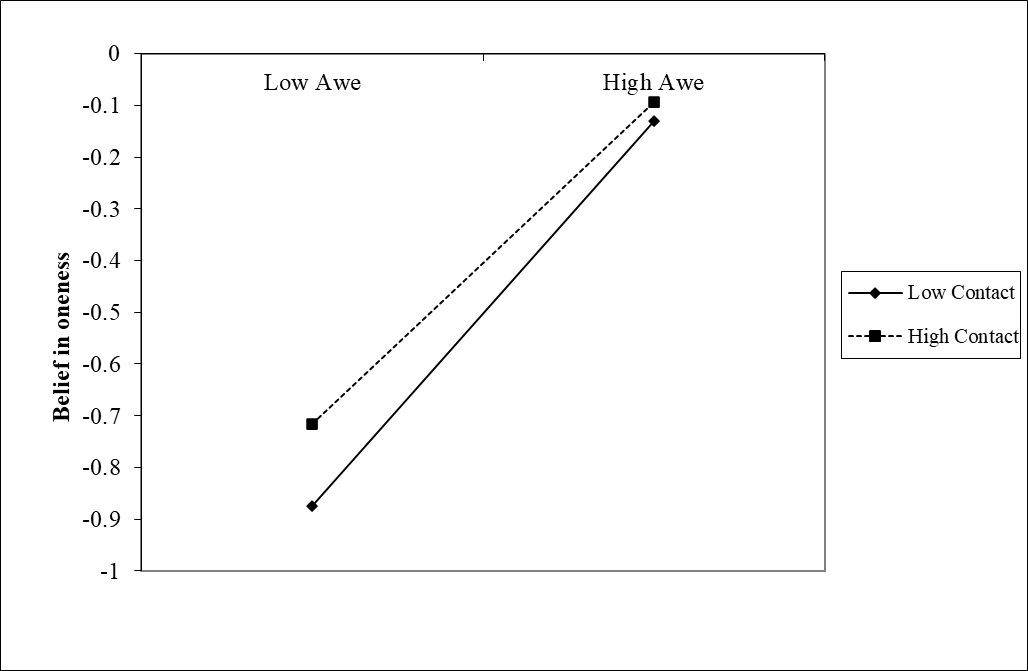


**Figure S8.** Decomposition of the interaction between awe and contact for different levels of contact (high = +1 *SD*; low = -1 *SD*), dependent variable: Prejudice (Study 3, LP1).

**
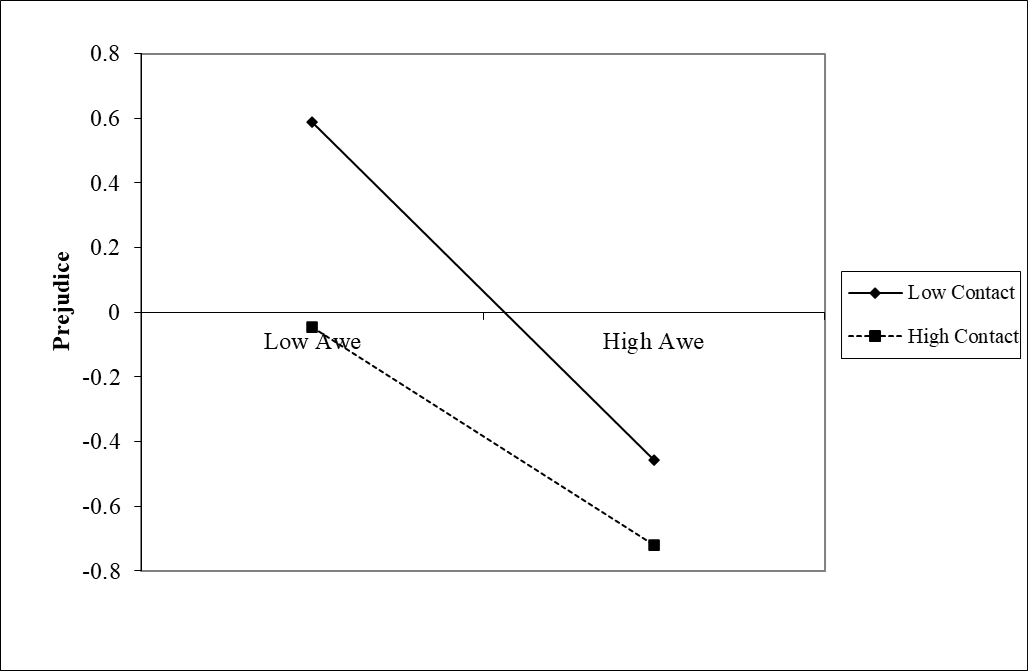
**

**Table S6**. Regressions conducted for the moderated mediation analysis—LP2 (Study 3).

| *Self-transcendence* | *R* | *R^2^* | *F* | *p* |
| --- | --- | --- | --- | --- |
|  | 0.46 | 0.21 | 42.74 | <0.001 |
|  | *β* | *SE* | *t* | *p* |
| Awe | 0.46 | 0.05 | 9.99 | <0.001 |
| Contact | 0.12 | 0.02 | 6.88 | <0.001 |
| Awe × Contact | −0.19 | 0.04 | −4.51 | <0.001 |
| *Belief in oneness* | *R* | *R^2^* | *F* | *p* |
|  | 0.47 | 0.22 | 45.78 | <0.001 |
|  | *β* | *SE* | *t* | *p* |
| Awe | 0.29 | 0.04 | 6.90 | <0.001 |
| Contact | 0.18 | 0.02 | 10.94 | <0.001 |
| Awe × Contact | −0.16 | 0.04 | −4.20 | <0.001 |
| *Prejudice* | *R* | *R^2^* | *F* | *p* |
|  | 0.45 | 0.20 | 26.83 | <0.001 |
|  | *β* | *SE* | *t* | *p* |
| Awe | −0.25 | 0.05 | −4.91 | <0.001 |
| Self-transcendence | −0.32 | 0.04 | −7.19 | <0.001 |
| Belief in oneness | 0.03 | 0.05 | 0.58 | 0.56 |
| Contact | −0.04 | 0.02 | −2.20 | <0.05 |
| Awe × Contact | 0.06 | 0.04 | 1.45 | 0.15 |
| General positivity disposition | 0.04 | 0.03 | 1.61 | 0.11 |

**Figure S9.** Moderated mediation model for LP2 in Study 3.

Belief in oneness

Awe

Prejudice

Self-transcendence

–0.32^***^

–0.25^***^

0.46^***^

Awe × Contact

0.03

0.29^***^

–0.19^***^

0.06

Contact

–0.04^*^

0.12^***^

0.18^***^

–0.16^***^

*Note.* Standardized coefficients are displayed for for the moderated mediation analysis-LP2; ^***^*p* < .001, ^*^*p* < .05.

**Figure S10.** Decomposition of the interaction between awe and contact for different levels of contact (high = +1 *SD*; low = -1 *SD*), dependent variable: Self-transcendence (Study 3, LP2).

**
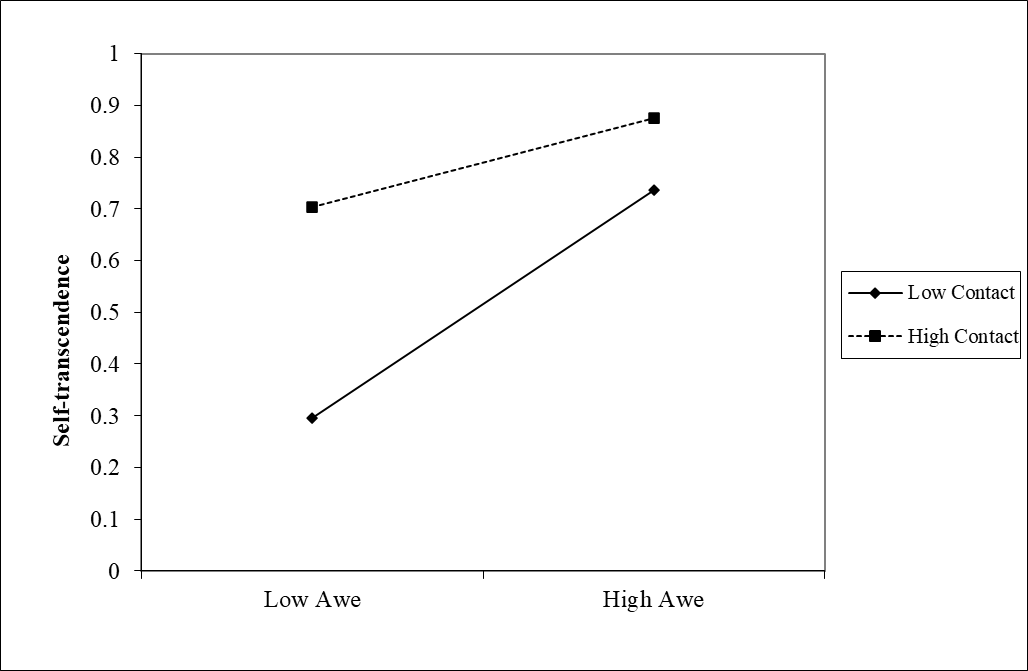
**

**Figure S11.** Decomposition of the interaction between awe and contact for different levels of contact (high = +1 *SD*; low = -1 *SD*), dependent variable: Belief in oneness (Study 3, LP2).


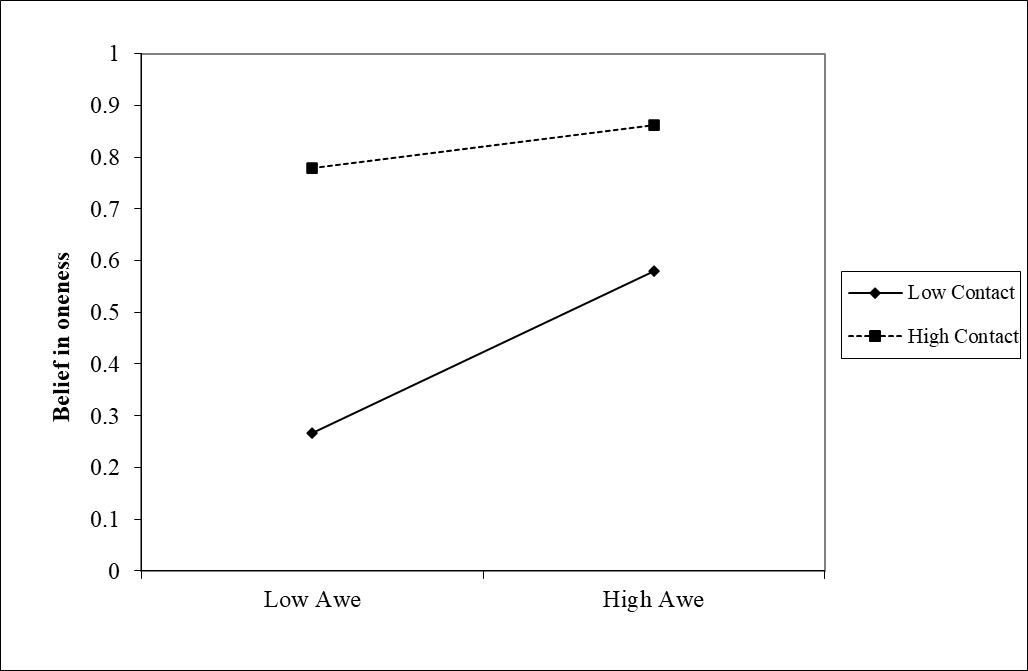


**Figure S12.** Decomposition of the interaction between awe and contact for different levels of contact (high = +1 *SD*; low = -1 *SD*), dependent variable: Prejudice (Study 3, LP2).

**
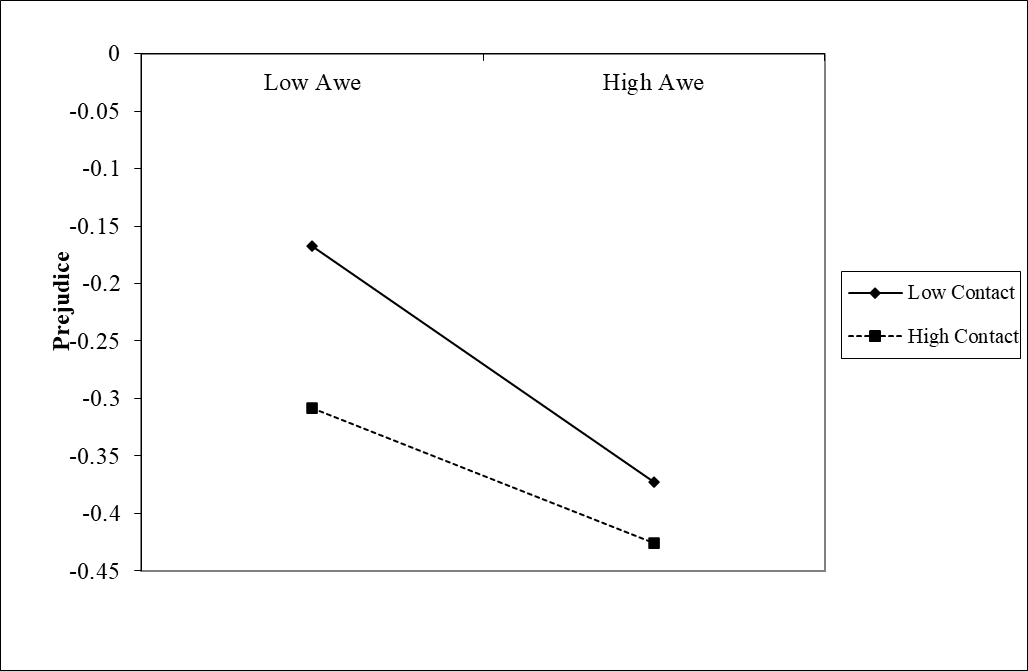
**

**References not present in the manuscript**

R Core Team (2023). *_R: A Language and Environment for Statistical Computing_. R* Foundation for Statistical Computing, Vienna, Austria. https://www.R-project.org/

Scrucca L., Fop, M., Murphy, T. B., & Raftery, A. E. (2016). mclust 5: clustering, classification and density estimation using Gaussian finite mixture models. *The R Journal 8*(1), 289-317. https://doi.org/10.32614/rj-2016-021

Wagenmakers, E. J., & Farrell, S. (2004). AIC model selection using Akaike weights. *Psychonomic bulletin & review*, *11*, 192-196. https://doi.org/10.3758/BF03206482
